# A novel network analysis approach reveals DNA damage, oxidative stress and calcium/cAMP homeostasis-associated biomarkers in frontotemporal dementia

**DOI:** 10.1371/journal.pone.0185797

**Published:** 2017-10-11

**Authors:** Fernando Palluzzi, Raffaele Ferrari, Francesca Graziano, Valeria Novelli, Giacomina Rossi, Daniela Galimberti, Innocenzo Rainero, Luisa Benussi, Benedetta Nacmias, Amalia C. Bruni, Daniele Cusi, Erika Salvi, Barbara Borroni, Mario Grassi

**Affiliations:** 1 Department of Brain and Behavioural Sciences, Medical and Genomic Statistics Unit, University of Pavia, Pavia, Italy; 2 Department of Molecular Neuroscience, Institute of Neurology, University College London (UCL), London, United Kingdom; 3 Department of Genetics, Fondazione Policlinico A. Gemelli, Roma, Italy; 4 Division of Neurology V and Neuropathology, Fondazione IRCCS Istituto Neurologico Carlo Besta, Milano, Italy; 5 Department of Neurological Sciences, Dino Ferrari Institute, University of Milan, Milano, Italy; 6 Department of Neuroscience, Neurology I, University of Torino and Città della Salute e della Scienza di Torino, Torino, Italy; 7 Molecular Markers Laboratory, IRCCS Istituto Centro San Giovanni di Dio Fatebenefratelli, Brescia, Italy; 8 Department of Neuroscience, Psychology, Drug Research and Child Health, University of Florence, Firenze, Italy; 9 Neurogenetic Regional Centre ASPCZ Lamezia Terme, Lamezia Terme (CZ), Italy; 10 Department of Health Sciences, University of Milan at San Paolo Hospital, Milano, Italy; 11 Institute of Biomedical Technologies, Italian National Research Council, Milano, Italy; 12 Department of Medical Sciences, Neurology Clinic, University of Brescia, Brescia, Italy; Biomedical Sciences Research Center Alexander Fleming, GREECE

## Abstract

Frontotemporal Dementia (FTD) is the form of neurodegenerative dementia with the highest prevalence after Alzheimer’s disease, equally distributed in men and women. It includes several variants, generally characterized by behavioural instability and language impairments. Although few mendelian genes (*MAPT*, *GRN*, and *C9orf72*) have been associated to the FTD phenotype, in most cases there is only evidence of multiple risk loci with relatively small effect size. To date, there are no comprehensive studies describing FTD at molecular level, highlighting possible genetic interactions and signalling pathways at the origin FTD-associated neurodegeneration. In this study, we designed a broad FTD genetic interaction map of the Italian population, through a novel network-based approach modelled on the concepts of disease-relevance and interaction perturbation, combining Steiner tree search and Structural Equation Model (SEM) analysis. Our results show a strong connection between Calcium/cAMP metabolism, oxidative stress-induced Serine/Threonine kinases activation, and postsynaptic membrane potentiation, suggesting a possible combination of neuronal damage and loss of neuroprotection, leading to cell death. In our model, Calcium/cAMP homeostasis and energetic metabolism impairments are primary causes of loss of neuroprotection and neural cell damage, respectively. Secondly, the altered postsynaptic membrane potentiation, due to the activation of stress-induced Serine/Threonine kinases, leads to neurodegeneration. Our study investigates the molecular underpinnings of these processes, evidencing key genes and gene interactions that may account for a significant fraction of unexplained FTD aetiology. We emphasized the key molecular actors in these processes, proposing them as novel FTD biomarkers that could be crucial for further epidemiological and molecular studies.

## Introduction

Frontotemporal Dementia (FTD), also known as Frontotemporal Lobar Degeneration (FTLD), is a neurodegenerative disorder characterised by deficit of executive functions, language impairment and behavioural disturbances [1,2]. FTD is considered the most common neurodegenerative dementia in the young adulthood along with Alzheimer Disease, and it is often familial (25–50%), usually with an autosomal dominant pattern of inheritance [3]. However, even though mendelian genetic determinants have been identified, such as microtubule associated protein tau (*MAPT*), Granulin (*GRN*) or *C9orf72*, in most of the cases no causative genes are recognized. For such cases, multiple loci and genes appear to influence FTD risk with rather small effect size [4].

Genome-wide association study (GWAS) is a powerful approach to evaluate the genetic components of human complex disorders including FTD [5–7]. The GWAS filtering strategy involves the evaluation of individual markers with the use of a genome-wide significance threshold p-value of 5*10^−8^ under the assumption of independence among markers. This approach minimizes false discoveries and was effective in uncovering multiple Single Nucleotide Polymorphisms (SNPs) associated with complex diseases and traits. However, the published GWASs for FTD relied on analyses at the SNP level with few reproducible and genome-wide significant findings, in independent samples [8]. To overcome SNP-based analysis limitations, gene-set analysis [9] has been proposed to examine groups of functionally related SNPs, grouped according to the corresponding gene locus. Gene-based tests range from the simple computation of overrepresentation of associated loci in annotation databases, including Gene Ontology (GO) biological processes (GO:BP) [10], KEGG [11] and Reactome [12] pathways up to the use of interaction networks and searches for subnetworks (modules) enriched with the associated genes [13–27].

Network-based models are more powerful than other methods, since they can simultaneously include different biological interactions, enabling a topology-aware gene prioritization and testing at different complexity scales, including single connections, pathways, and communities [13–18]. Moreover, mapping genetic data onto a reference interactome provides a straightforward and meaningful way to model gene context [28,29]. Typically, in network-based approaches, the context of a gene is thought to determine at least part of its properties. According to the so-called guilt-by-association rule, genes with a related function tend to be proximal in the interactome topology and share common profile patterns (e.g. gene expression levels). However, this may lead to misleading conclusions, especially for GWAS data, in which genetic variability may have subtle and unpredictable functional effects, close to statistical “noise” [8,19,29]. Three main reasons can be addressed through guilt-by-association under-performance: (i) the presence of hubs, being over-represented because attracting a large fraction of network interactions, (ii) the substantial topological difference between interaction networks and the directed acyclic graphs used to represent functional information in bio-ontologies, and (iii) compositional interactome biases due to well-studied groups of genes that may cause artificial over-representation of their associated terms. By contrast, a recent study [29] suggests that few biologically-critical interactions may account for a large fraction of the functional information content of the entire network, that do not necessarily involve hubs.

**Fig 1:**
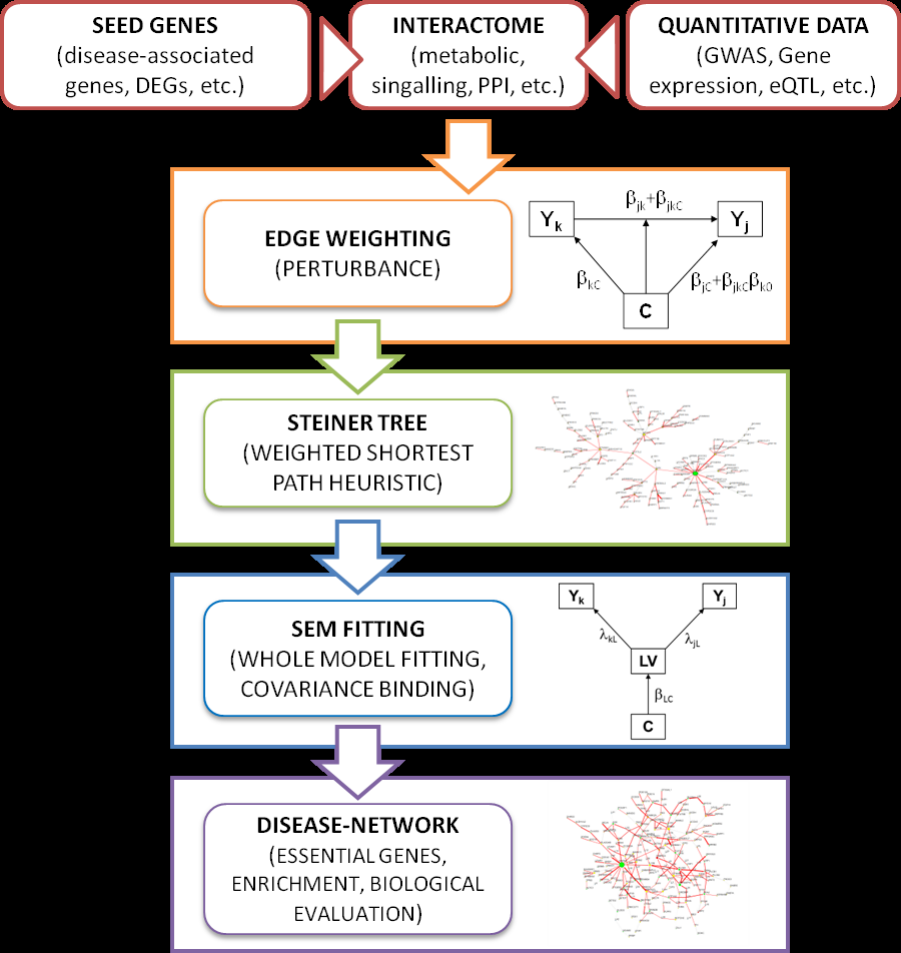
Data analysis workflow. Our network-based data analysis method includes three inputs: (i) a reference interactome that is used as a gene-gene interaction space; (ii) a set of seed nodes representing terminals (sources and targets) of information spreading through the interactome; and (iii) quantitative data used to build network weights. Weights are then used to generate a Steiner tree connecting seed genes through paths maximizing edge perturbation, using a weighted heuristic shortest path algorithm. The resulting Steiner tree is then converted into a Structural Equation Model (SEM) and fitted, to assess its validity. During SEM-based procedure, covariance between pairs of leaf genes (i.e., ancestral bow-free nodes) are tested and fitted using a latent variable (LV) model. The group variable *C* = {0, 1} influences a LV, modelling the unobserved cause(s) acting on the two target genes. Significant covariances are retained in the extended network, representing the final disease-network.

In the present work, we applied a novel network-based method that combines the principles of connectivity significance [13–17], through the concept of node and edge perturbation [18]. Genetic information encoded in the GWAS data was used to select seeds and weight the differential gene-gene co-variation, to reduce the network to its essential parts, removing non-informative noise and misleading nodes. To this end, we performed Steiner tree search [30–33], combined to Structural Equation Model (SEM) analysis [34–36]. Furthermore, we know that complex disorders tend to form specific sub-network structures, corresponding to groups of perturbed disease-associated genes [13–18,28,29]. We considered module sub-structure to characterize disease local perturbation, based on significant genetic differences between cases and controls. Through this molecular network framework, we built a metabolism-based FTD pathogenesis theory that can be summarized in two main steps: (i) oxidative damage accompanied by loss of neuroprotection, and (ii) abnormal neuronal activity and neurodegeneration. In the following discussion, we will examine the molecular bases of both processes, proposing a root for further molecular investigations in FTD aetiology. The goal of our pipeline, shown in Fig 1, was to extend the list of FTD risk genes uncovering the functional network underlying sporadic (i.e., non-mendelian) variability associated with this complex disorder.

## Materials and methods

### Ethics statement

#### Case data

Investigators at every site obtained written informed consent from patients and control individuals [4,6]. Every participating group provided consent to use the samples for the purposes of this study. Each study site obtained approval from a local ethics committee (UK ethics committee number 10/H0716/3) or institutional research ethics board.

#### Control data

Control data [37] presented in this work are part of the HYPERGENES Project (European Network for Genetic-Epidemiological Studies; www.hypergenes.eu). Institutional review boards at each collection site approved the study and all individuals gave their informed consent. A further Ethical Revision of the University of Milano and of the HYPERGENES Internal Ethical Steering Board approved the entire process.

### Input data

Our network-based analysis relies on three inputs: (i) quantitative data, (ii) a set of seed genes, and (iii) a reference interactome.

The GWAS data used as input in the current work were generated in [4]. There the features of the study population were described in detail. For the cases, genotyping data of DNA samples diagnosed with FTD were available from the International FTD-GWAS data set for 634 samples obtained from 8 Italian research centers. After quality check (QC) steps 530 patients diagnosed with bvFTD (n = 418), semantic variant PPA (n = 27), agrammatic variant PPA (n = 61), and FTD-MND (n = 23) survived. All cases were diagnosed according to the Neary criteria [38] and/or the more recent Rascovsky and Gorno-Tempini criteria [1,2]. Age of onset was 64.1 ± 20.7 years (Mean ± SD); range: 29.0–87.0), including 287 (54.2%) women. The cases were collected and genotyped at the University College London by means of Illumina human 660K-Quad Beadchips assayed on the Illumina Infinium platform (Illumina, San Diego, CA, USA).

For the controls, genotyping data were obtained from the HYPERGENES project (European Network for Genetic-Epidemiological Studies; www.hypergenes.eu) [37]. All participants in the sample set (n = 1327; 926 after QC) had no abnormal findings on physical and neurological examination, were unrelated, collected in Italy, and of Caucasian ancestry. Age was 58.2 ± 6.1 years mean (±SD); range, 50.0–97.0), including 349 (37.7%) women. The control samples were genotyped at the University of Milan, using the Illumina 1M-duo array.

Quantitative data was generated by applying supervised Principal Component Analysis (sPCA) [39] over additive-encoded genotypes (0 for the frequent homozygote genotype, 1 for the heterozygote, and 2 for the rare homozygote genotype), and taking the first principal-component score (PC1) for each gene, as reported in our previous study [4]. Briefly, polymorphisms were grouped by gene membership, where the gene region is defined by its locus coordinates ±5000 base pairs. A single gene was then represented as a continuous score by taking the first supervised PC1 on a subset of SNPs selected using outcome (case-control) information, i.e. supervised PC1 was a weighted sum of the SNPs within the gene region that maximize the variance of the gene score, which then varies with the outcome.

Seeds are genes of interest, that can arbitrarily be chosen using existing knowledge (e.g. from databases such as OMIM, DisGeNET, or by text mining), and/or experimental data analysis (e.g. GWAS, expression data, or DNA-binding data). We used the list of FTD-associated genes from our previous SNP-to gene approach [4], applying an FDR threshold < 10%, yielding 280 putatively FTD-associated genes. These genes were referred to as the FTD-seeds.

An interactome is defined as the ensemble of known interactions among biological entities (e.g, genes, proteins, or metabolites) in a given organism, usually represented as a network. The interactome can be retrieved from many different sources, including KEGG [11], Reactome [12], STRING [40], GeneMANIA [41], MINT [42], IntAct [43], BioGRID [44]. We selected KEGG, for different biological and computational reasons, including annotation curation and completeness, and GO biological process mappability for functional annotation. Furthermore, KEGG is a directed interactome, thus causality can be easily interpreted in terms of biological signalling, and can be used for the FTD model validation and extension. However, edge direction is not a necessary requirement in our method. We retrieved KEGG signalling pathways and merged them to obtain a unique network using graphite [45] R software package.

### Network edge weighting

The input interactome was converted into a weighted network, endowed with node and edge weights reflecting their perturbation status. Genes (nodes) were weighted as being FTD-seed (weight = 1) and non-seed (weight = 0). Gene-gene interactions (edges) were weighted based on the case/control statistical difference. In our notation, *j* and *k* represented any two connected nodes of the network, with *j->k* being the edge direction. In general, we tested if the total difference between case vs. control groups for gene *k* through gene *j* was significant, given data. This implied testing the group change at the same time on gene *j*, gene *k* and their direct link *j->k*. The p-value was yielded by a t-test on the combined difference of the group over the node *j*, the node *k*, and their direct connection *j->k*, fitting a trivariate (*X* = *j*-th gene, *Y* = *k*-th gene, *C* = {0, 1}) Structural Equation Model (SEM) via lavaan R package [46].

Then, the edge weights were defined as inverse of negative logarithm of the p-values, *w* = 1/-log(p-value). In this way, edges with lower p-values had lower weights, on a positive continuous range. Intuitively, this weight can be assumed as the perturbance acting on the relationship between two connected genes in the interactome, due to the genotype difference between groups. The lower the p-value (i.e. the weight), the higher the perturbance. In general, we defined the perturbance over a node *k*, due to the action of a node *j*, as the altered status of *j* and *k* genes, and their *j->k* interaction in the diseased sample, comparatively to healthy controls.

### The Steiner tree problem

The FTD seeds were mapped to the weighted interactome, and a FTD-related sub-network was constructed by adding new genes to connect FTD genes solving a Steiner tree problem [30–33], minimizing the sum of weights of every edge in the sub-graph. We applied a modified shortest path heuristic (SPH) distance algorithm, from Kou *et al*. solution [33], implemented in our subnet() R function. Our algorithm selected outgoing shortest paths combing the edge weights by Fisher’s method and testing the statistical significance (p < 0.05) with multiple comparison Bonferroni correction. The resulting Steiner tree, corresponding to the maximum-perturbance sub-graph, preserves the original directed edges. Therefore, we distinguish three types of nodes: “*sources”* (emitting perturbance) with no incoming connections, “*targets”* (absorbing perturbance) with no outgoing connections, and *“connectors*” (transmitting perturbance) with incoming and outgoing connections. We referred to a perturbation route, as a perturbed path originating from a source node, traversing a number of connectors, and terminating in a target node. This tree was used as a backbone for the subsequent augmenting SEM step.

### Structural Equation Model (SEM) analysis

The sub-graph obtained as a Steiner tree was converted into a SEM [34–36], such that every node in the sub-network corresponds to a variable of the SEM, and every edge is a relationship between variables. In summary, a group node (*C* = {0, 1}) connected to each gene was added inside the Steiner tree, and the overall sub-graph was converted into a system of linear equations, and then fitted using PC1-trasformed SNP genotype data. The system of linear equations has the form:

$$\begin{array}{l} Y_{j}=\beta_{jC}C+U_{j};\;j\in V(x)\\ Y_{j}=\beta_{jC}C+\sum_{k\in pa(j)}\beta_{jk}Y_{k}+U_{j};\;j\in V(y) \end{array}$$

with a covariance structure:

$$cov(U_{j};U_{k})=\left\{ \begin{array}{l} \psi_{jk}\;\mathrm{if}\;j=k\;\mathrm{or}\;j\in sib(j)\\ \;0\;\mathrm{otherwise} \end{array}\right.$$

where V(x) and V(y) are, respectively, the sets of the exogenous variables (i.e. source genes) and endogenous variables (i.e, connector plus target genes) in the sub-network. The linear equations define the relationships between the variable *Y*_*j*_ with the group variable *C* and variables *Y*_*k*_ in the “parents” set, *pa (j)*, quantified by path coefficients β_jC_ and β_jk_, respectively. Unobserved variables *U*_*j*_ represent the variation of each *Y*_*j*_ not explained by its parent nodes. The covariance structure describes the bi-directed relationships between variables in the “siblings” set, *sib (j)*, quantified by covariances *ψ*_*jk*_, and interpreted as unmeasured common causes for pairs of variables.

SEM analysis allowed to evaluate: (i) the overall goodness of fit of alternative constrains in the models, (ii) the significance of the group difference on every gene, and (iii) how significant the covariance between target nodes (i.e., genes that are not connected by a directed path) was, added to improved SEM goodness of fit. The significant new edges added in (iii), called “ancestral bow-free” covariances in SEM literature closed the directed paths into circuits characterized by the presence of signaling sources and targets.

Minimun AIC (Akaike Information Criterion) score, and a standardized root mean squared residual (SRMR) less than 0.05 were considered for model selection and overall good model fitting, respectively [34]. The statistical significance of the SEM parameters (the regression coefficients and the ancestral bow-free covariances) were estimated by Maximum Likelihood Estimation (MLE) and the beta coefficients were evaluated through t-test with bootstrap standard error (SE) with B = 1000 resamplings, and the significance level established at p < 0.05, two-sided.

After Fisher’s normalizing t-transformation of the covariance, ancestral bow-free covariances, with abs(t) > 2 were selected. Successively, the selected covariances were tested by a SEM with Latent Variables (LVs) [47] using a model in which two target genes are connected through a LV modelling the underlying common unknown cause(s) acting on them. Every LV is subjected to group (*C* = {case, control}) effect. In this context, a LV is a variable not present in the initial Steiner tree, introduced to capture the differential case/control co-variance between two disconnected target nodes. The goodness of fit of each LV model was evaluated through a Likelihood Ratio Test (LRT), where a good fit corresponds to a p-value > 0.05 (i.e., there is no significant difference between sample and model covariance matrices). The influence of the group *C* over each LV was evaluated by a t-test (= MLE/bootstrap SE) with p-value < 0.05, two sided. Every step of the SEM analysis was performed calling the lavaan [46] and igraph [48] R packages in our sem2group() R function.

### Functional analysis

After model building SEM assessment and model extension with LVs underling covariances between pairs of leaf genes, we proceeded with the biological evaluation. To improve the FTD model interpretation, we initially focused on nodes with specific topological properties (e.g., those connecting different portions of the gene network). We computed various topological indices, including in/out degree, i.e. the number of incoming/outgoing connections for a node, and weighted node betweenness [28], i.e. the number of weighted shortest paths travelling through a node in the network, by means of perturbance. Using these topological indices, we identified four types of nodes: (i) “springs”, i.e. source nodes with high out-degree. (ii) “sinks”, i.e. target nodes with high in-degree; (iii) “hubs”, i.e. connector nodes with high in/out-degree, and (iv) “bottlenecks”, i.e. nodes with high weighted betweenness. We also defined as secondary sources many direct targets of proper sources, with in-degree 1 and out-degree 1, representing ligand-receptor interactions.

We focused on the concept of “essential” node, by combining definitions (iii) and (iv) as critical nodes for the FTD-network integrity, and part of the network backbone. These nodes carry the majority of perturbed information flow through the FTD network, and therefore we expect them to be assumed as suitable novel FTD risk factors.

Annotation enrichment over the main biomedical ontologies was used to functionally validate and extend our biological interpretation of the FTD model. Enrichment analysis was performed using a hypergeometric test with Bonferroni correction (adjusted p-value < 0.05), over GO:BP [10], KEGG [11], Reactome [12], and Disease Ontology (DO) [49]. Network indices and Enrichment analysis were calculated calling the igraph [48], clusterProfiler [50] and DOSE R packages [51].

## Results

### The FTD sub-network

We first generated the SNPs-to-Genes data set, taking the first principal-component score (PC1) for each gene obtained by applying supervised Principal Component Analysis over additive-encoded genotypes (see Materials and Methods). We then used the 280 FTD-associated genes from our previous study [4] as seed list, applying a False Discovery Rate (FDR) threshold < 10%. We also compiled the reference human interactome by merging KEGG signalling pathways [11]. After mapping PC1 quantitative data (13971 genes) over the KEGG database, our interactome was made of 4073 nodes and 34606 weighted edges (23075 directed and 11530 bidirected), with a median vertex degree of 4 and average directed shortest path distance equal to 5.464. Finally, gene-gene interactions (i.e. edges) were weighted by combining the effects of the group variable (i.e. *C* = {0, 1}, representing controls and cases, respectively) over pairs of nodes, and their direct connection (see Materials and Methods).

The interactome contained 83 out of 280 FTD seeds, with a median vertex degree of 7 and average directed shortest path distance equal to 4.396. Notably, a higher vertex degree in combination with a shorter weighted shortest path for the mapped FTD seeds showed that FTD-associated genes are more clustered than the rest of the interactome. To assess the significance of this finding we tested if the mapped FTD-seeds were more connected than a random selection of 83 genes from the interactome (B = 1000 samples) or a node permutation of the interactome (B = 1000 permutations). Results showed that there was a significantly higher degree (p = 0.034 and p = 0.029 for randomization and permutation tests, respectively) and a significantly shorter average shortest path (p < 0.001 for both tests) for the FTD genes.

**Fig 2:**
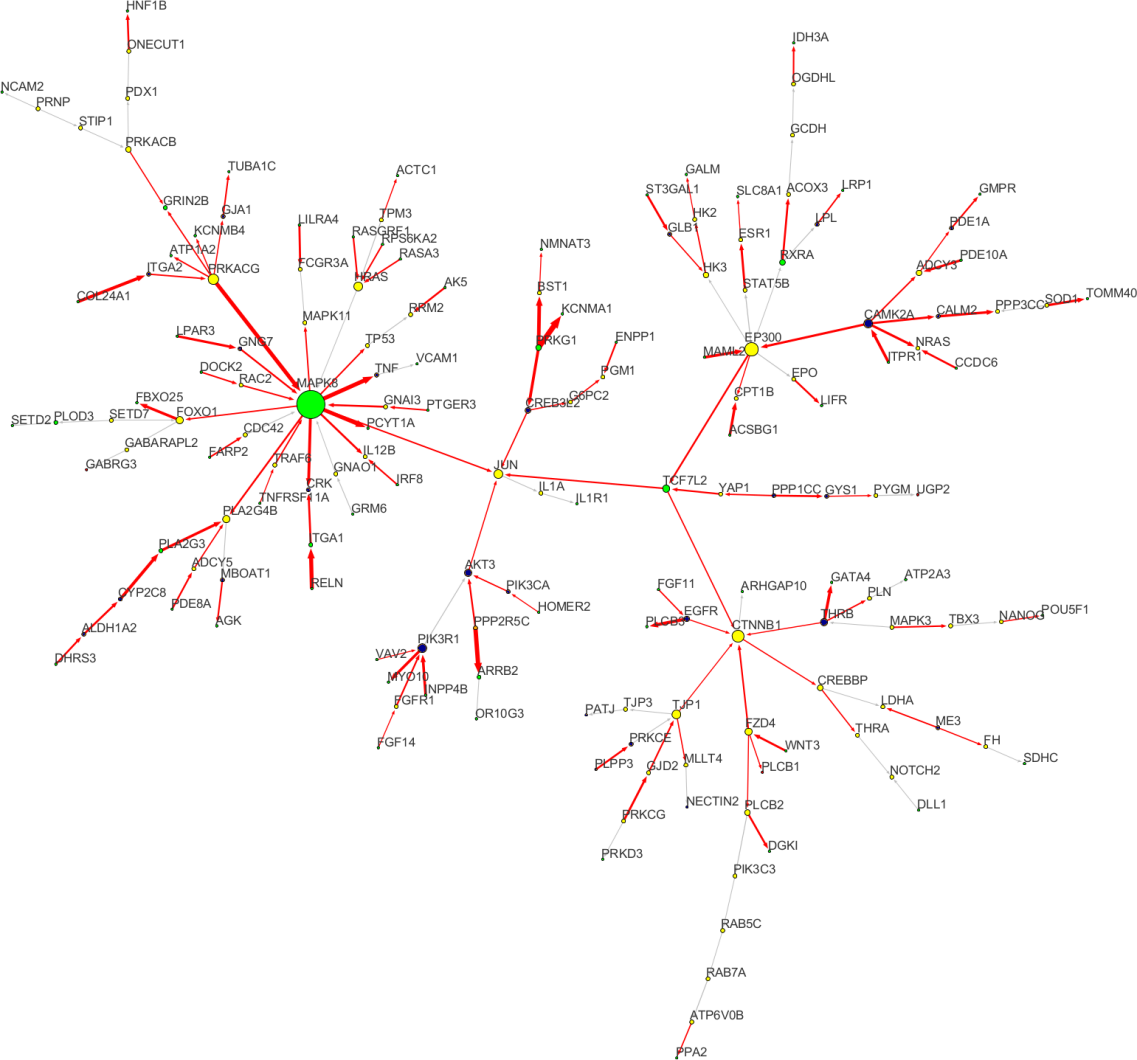
Steiner tree. Steiner tree obtained applying a Shortest Path Heuristic (SPH) algorithm. The tree has 167 nodes and 166 edges. The size of each node (i.e. gene) is proportional to its degree (i.e. the number of incoming and outgoing connections). Node colours indicate: perturbed seeds (green), non-perturbed seeds (red), perturbed connectors (blue), and non-perturbed connectors (yellow). Red edges correspond to perturbed interactions, while edge thickness is proportional to their weight (i.e. their perturbance level). A perturbed interaction has a weight *w* < 0.33 (i.e. p < 0.05 threshold over the nominal p-value). The entire network is characterized by a backbone *CAMK2A-TCF7L2- CTNNB1-JUN-MAK8-PRKACG*, where *CAMK2A* and *MAPK8-PRKACG* are the main perturbed hubs, *CTNNB1* and *JUN* represent the sinks of the entire system, and *TCF7L2* is the bottleneck connecting them.

We applied the Steiner tree algorithm to the mapped FTD seeds, detecting an initial set of 3854 shortest paths between the 83 seeds. The final FTD Steiner tree (Fig 2), including only connector genes traversed by perturbed paths, contained 167 nodes (77 out of 83 FTD seeds) and 166 edges (117 of which significantly perturbed, p < 0.05). The Steiner’s tree topology revealed the presence of: (i) 43 sources, of which 33 FTD seeds; (ii) 49 (38 FTD seeds) targets; and (iii) 75 (6 FTD seeds) connectors. The Steiner’s tree backbone, defining the mainstream perturbation route, was defined by the genes *CAMK2A*-*EP300*-*TCF7L2*, bifurcating to *EGFR*-*CTNNB1* and *MAPK8*-*JUN* (Fig 2).

SEM analysis of the Steiner tree was performed by fitting various models with different constraints on their parameters; the selected model, with fixed beta coefficients, equal variances, and bow-free covariances, yielded the lowest AIC score (AIC = -9324) and good fitting (SRMR = 0.026; see Table 1).

**Table 1:** SEM goodness of fit. Goodness of fit measures for different models, fitted to multivariate data of the extracted Steiner tree. The selected model, indicated by (*), has the lowest Akaike Information Criterion (AIC = -9324.98).

MODEL	*t*	LRT	df	AIC	BIC	SRMR	Elapsed Time
**Beta = ADE, equal variances, zero covariances**	169	22172.34	14027	-5881.66	-80011.8	0.028	5.73473 sec
Beta = ADE, equal variances, bow-free covariances (*)	1444	16179.02	12752	-9324.98	-76717	0.026	25.6701 min
**Beta = ADE, unequal variances, bow-free covariances**	1610	16178.6	12586	-8993.4	-75508.2	0.026	26.3120 min
**Beta = {*a*, *b*}, equal variances, bow-free covariances**	2334	15588.33	11862	-8135.67	-70824.2	0.027	21.8453 min
**Beta = {0.1, -0.1}, equal variances, bow-free covariances**	2332	17595.51	11864	-6132.49	-68831.6	0.028	33.4079 min
**Beta = {0.1, -0.1}, unequal variances, bow-free covariances**	2492	17583.6	11704	-5824.4	-67677.9	0.029	22.0544 min
**Estimated Beta, equal variances, bow-free covariances**	1610	15988.02	12586	-9183.99	-75698.7	0.026	23.8375 min

ADE = Average Direct Effect of the *k*-th node on the *j*-th node, weighted by group frequencies; *t* = number of model parameters; LRT = model likelihood ratio test; df = model degrees of freedom; AIC = Akaike Information Criterion; BIC = Bayesian Information Criterion; SRMR = Standardized Root Mean Squared Residual; Elapsed Time is calculated as CPU running time using an HP workstation with 24GB of memory and dual CPU (8 core) Intel Xeon X5570–3

Non-parametric bootstrap estimate of the SEM beta coefficients (B = 1000 samples with replacement) detected 98 significantly perturbed genes (bootstrapping p-values < 0.05), of which 74 FTD seeds, indicative of association with the disease (S1 Table). After Fisher’s normalizing t-transformation of the covariance, 43 ancestral bow-free covariances, connecting 43 (35 seeds) target genes (S2 Table) with abs(t) > 2 were selected. Adding the latent variables (LVs) underlying covariances between pairs of target genes, we obtained a sub-network of 210 (167 genes + 43 LVs) nodes and 252 (166 + 2*43) edges (S1 Fig). Among the 43 LVs, 20 yielded a good LV-model fit and resulted as significantly perturbed (i.e., LV average values are different in cases respect to controls), having bootstrapping p(C->LV) < 0.05 and p(LRT) > 0.05 (S2 Table).

### Topological analysis and essential nodes

To drive and improve our biological interpretation of the FTD network, we initially focused on nodes with specific topological properties, their function, and their possible implication in neurodegeneration. In particular, we searched for those genes in the FTD model that connect different perturbed routes throughout the network. Hereafter, we will use “essential nodes” to designate a class of genes that share a high number of perturbed connections (especially outgoing ones), thus bearing the largest amount of perturbed information spreading through the FTD network. This definition differs from the usual definition of hub, since it takes into account both the number of perturbed connections and perturbed shortest paths traversing a given node (Fig 2 and S1 Fig). Many of these are terminal seed-genes (i.e. peripheral nodes), designed as FTD-associated in our previous analysis [4] and confirmed as perturbed in the present study (i.e. green nodes in Fig 2 and S1 Fig). Besides seeds, we also identified additional FTD-associated genes (blue and red nodes in Fig 2 and S1 Fig), using SEM testing. However, also non-perturbed genes (i.e. not carrying FTD-associated variants) are functionally altered if they are significantly influenced by genes carrying disease-associated variants (i.e. they have perturbed incoming connections). Based on the perturbation routes, we could define topologically-critical FTD-associated genes.

**Fig 3:**
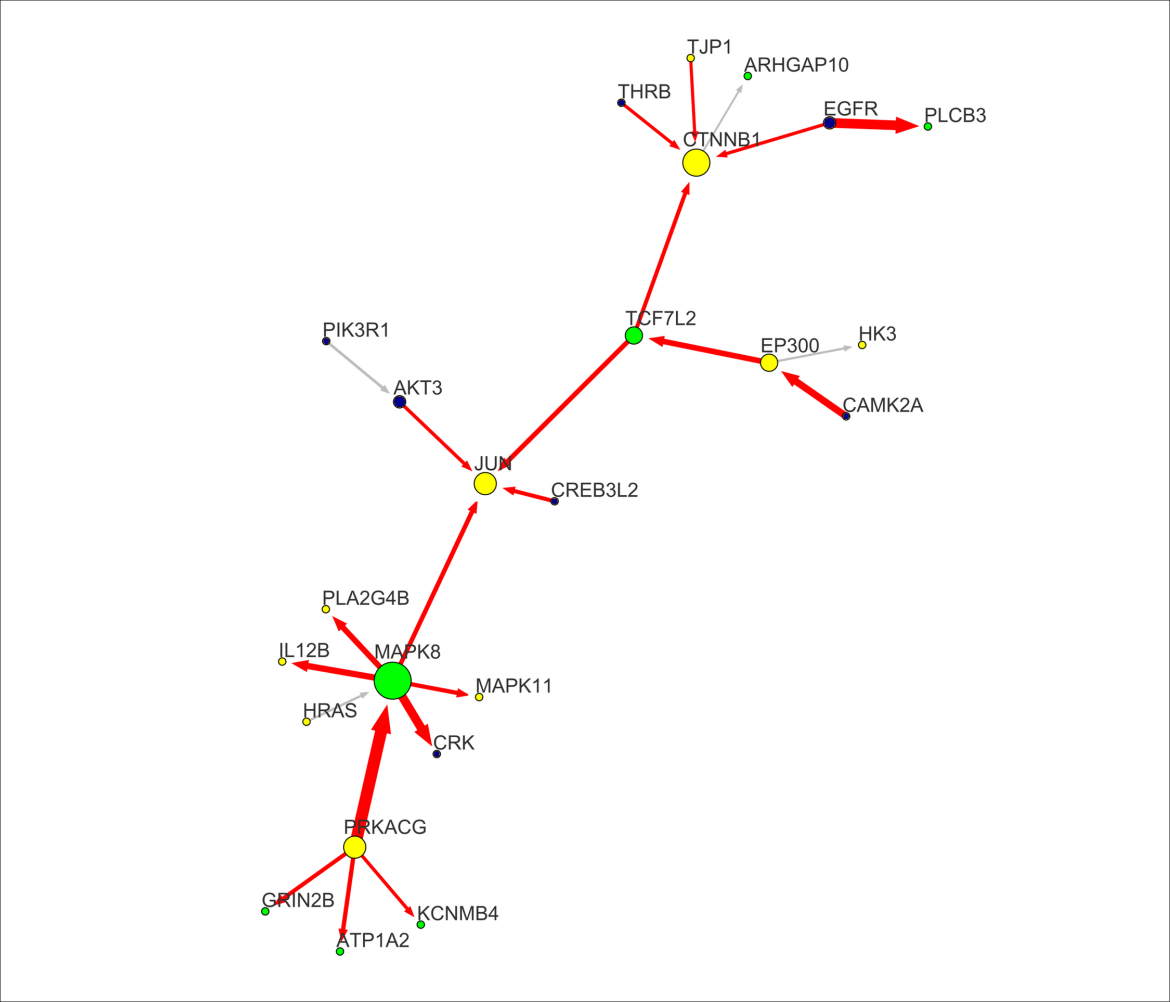
Essential node sub-network. Node essentiality is determined by considering nodes having both degree centrality and weighted betweenness centrality over the upper-quartile. Essential nodes are placed in non-redundant portions of the network and thus cannot be removed without a deep impact on network connectivity. These genes intercept the network backbone, represented by the axis *TCF7L2-JUN-MAK8-PRKACG*, carrying the top perturbation levels, especially in proximity of the sources. Nodes and edges are labelled according to the conventions followed in Fig 2.

A set of connectors bind sources to targets. These genes can both receive and send perturbed interactions. Steiner’s connectors are genes with generally high centrality. They may have high degree (i.e. hubs, with degree > 3), such as *MAPK8*, and/or high betweenness (i.e. they are important for connecting network modules), such as *TCF7L2*. Connectors are key genes revealing the set of critical trait-specific molecular processes for the cell. Among all nodes in the FTD-module, the most central ones are designed as essential. Essential nodes are those genes that cannot be removed without a deep impact on network connectivity. Considering that the FTD network has been generated based on phenotype-associated genotypic variability, essential nodes should be also functionally critical for the FTD phenotype. To point out the most central connectors, we extracted an essential high weighted-betweenness sub-network (Fig 3). Essentiality combines the concepts of biological process perturbation with the importance for network structural integrity. The essential sub-network defines the FTD-network backbone, characterizing the most perturbed interactions. They include: (i) the *EGFR*-*PLCB3* interaction, (ii) the *CAMK2A*-*EP300*-*TCF7L2* perturbed path, and (iii) the *PRKACG*-*MAPK8* interaction. The essential backbone highlight the presence of nodes with exceptionally high outgoing connectivity (hubs), including *MAPK8* and *PRKACG*, and exceptionally high incoming connectivity (i.e., sink nodes), including *CTNNB1* and *JUN*. Sinks of perturbation represent nodes with no outgoing perturbed interactions, that ideally encompass the model terminal functions, including (post)synaptic plasticity and cell death (see Discussion).

**Fig 4:**
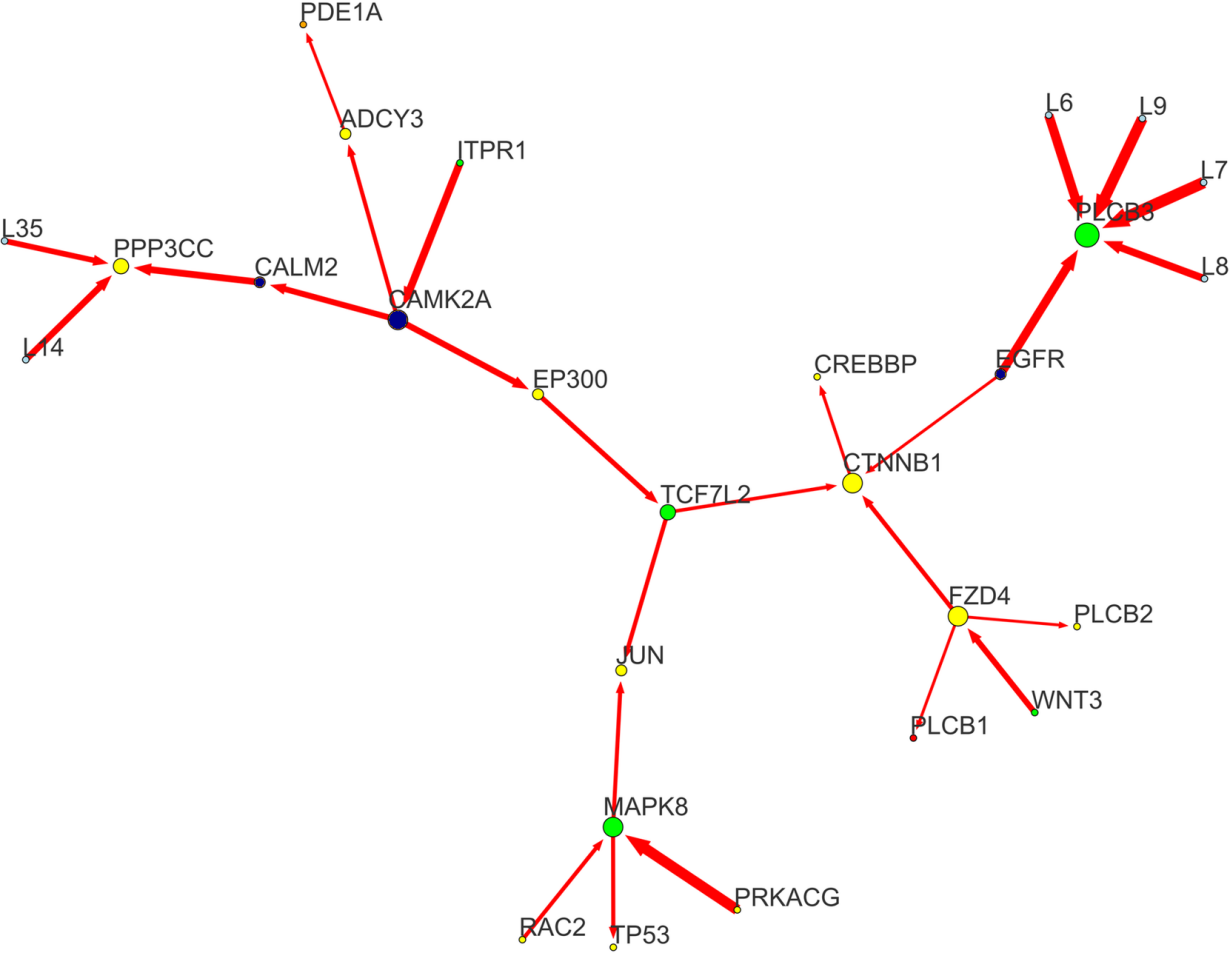
ncWNT sub-network. This sub-network focuses on the module characterized by a series of receptors and enzymes regulating Calcium/cAMP homeostasis and involved in the non-canonical Ca^+2^/WNT signaling pathway. Among them, the most perturbed are the *EGFR* receptor and its target phospholipase *PLCB3*, and the routes *ITPR1-CAMK2A-CALM2-PPP3CC* and *ITPR1-CAMK2A-EP300-TCF7L2*. *JUN* is a large sink between this module and the MAPK-JNK one. Nodes and edges are labelled according to the conventions followed in Fig 2.

**Fig 5:**
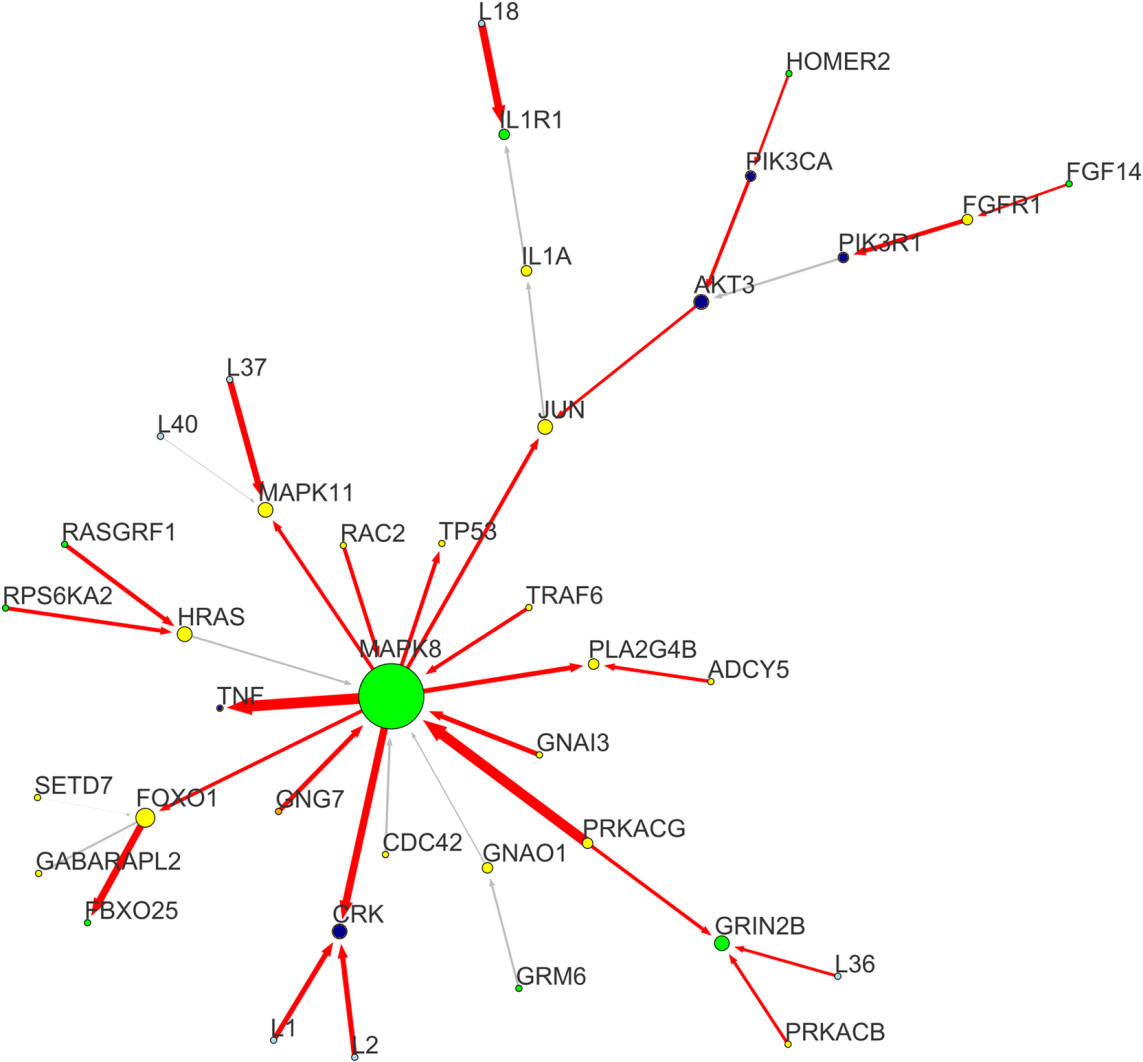
MAPK-JNK sub-network. This sub-network focuses on the module characterized by a series Serine/Threonine kinases involved in the MAPK-JNK signaling pathway. Among them, *MAPK8* is the one having the highest outgoing connectivity of the entire network, and the most perturber incoming interaction carried by *PRKACG*, another FTD-network hub. Other deeply perturbed interactions include *MAPK8-TNF*, *MAPK8-CRK*. *JUN* is a large sink within this module and the non-canonical WNT pathway (ncWNT) one. Nodes and edges are labelled according to the conventions followed in Fig 2.

Special attention must be given to unobserved variability, represented by LV-mediated interactions (Figs 4 and 5 and S1 Fig). Although latent (i.e. not explicit in the model), LV-associated variability may evidence common causes of perturbation. Considering LVs only, *PLCB3*, a phospholipase C (PLC) controlled by *EGFR* in our model, is the sink node with the highest incoming perturbance level (Fig 4). This is particularly interesting as this gene might be responsible of Ca^+2^ homeostasis impairments in FTD (see Discussion). Remarkably, *PLCB3*-specific LVs (LV6-9) connect this gene to four FTD-relevant targets: (i) *GABRG3*, a perturbed/seed GABA receptor, the major inhibitory neurotransmitter in mammalian brain; (ii) *IL12B*, an osmotic stress-induced cytokine, targeted by *MAPK8*; (iii) *DGKI*, a DAG-kinase interacting with the phospholipase *PLCB2*; and (iv) *ARHGAP10*, a GTPase activator involved in apoptosis. Other perturbed LVs connections (Figs 4 and 5) include: *CRK*, *AGK*, *ATP2A3* (LV1-2); *IL1R1*, *CPT1B* (L18); and *PPP3CC*, *VCAM1*, *PRKD3* (LV14 and LV35). S1 Fig reports the whole gene and LVs connectivity in the FTD-network.

### Enrichment analysis

The enrichment analysis was performed on the whole set of nodes of the FTD-network. Enrichment analysis over GO:BP, KEGG, and Reactome showed significant involvement of response to oxygen-containing compounds (ROCC), immune response, and homeostatic processes (S2 Fig), several signaling pathways, including MAPK, RAS, cAMP/cGMP, the WNT pathway, and long-term potentiation (LTP) (S3 Fig), and synaptic transmission-related processes (S4 Fig), including EGFR/FGFR signaling, transmission across chemical synapses, NMDA receptor-associated postsynaptic activation, phospholipase C-mediated cascades, and Calcium pathway. Furthermore, Disease Ontology (DO) data showed enrichment for nervous system and mental health-related terms, including: central nervous system disease, cognitive disorder, psychotic disorder, and neurodegenerative disease (S5 Fig).

The most significant term in GO:BP is the *response to oxygen-containing compound* (ROCC; GO:1901700; p < 1E-9), involving the majority of source nodes and their targets, suggested that oxygen compounds and reactive oxygen species (ROS) may trigger the whole network perturbation. Particularly, in the FTD-network (Fig 2 and S1 Fig) oxidative stress was impacted due to impaired energetic metabolism [52,53] eliciting the activation of stress-responsive receptors, including Frizzled-4 (*FDZ4*) and *EGFR* [54–64], and oxidative stress-inducible Serine/Threonine kinases *CAMK2A*, *PRKACG*, *PRKACB*, and *PRKG1* [54–83]. Collaterally, these effectors may further induce neuron membrane potentiation, leading to apoptosis through activation of the MAPK-JNK signaling pathway [73–77] and Ca^2+^/cAMP metabolism deregulation [84–91]. Aberrant Ca^2+^/cAMP metabolism might lead to anomalous neuron potentials and further deficits in neuroprotection, neuron functioning, and survival [69,70,80–100].

More specifically, the significant KEGG-enriched (S3 Fig) and Reactome-enriched (S4 Fig) functions sustained by the FTD-network, included: *MAKP signaling* (KEGG:map04010), *RAS signaling* (KEGG:map04014), *cAMP signaling* (KEGG:map04024), *cGMP-PKG signaling* (KEGG:map04022), *Gap Junction* (KEGG:map04540), *WNT signaling* (KEGG:map04310), *Insulin signaling* (KEGG:04910), *Long-Term Potentiation* (KEGG:map04720), and *FoxO signaling* (KEGG:map04068), *Signaling by EGFR* (Reactome: R-HSA-177929), *DAP12 signaling* (Reactome: R-HSA-2424491), *Transmission across chemical synapses* (Reactome: R-HSA-112315), *Neurotransmitter receptor binding and downstream transmission in the postsynaptic cell* (Reactome: R-HSA-112314), *G protein mediated events* (Reactome: R-HSA-112040), *PLC beta mediated events* (Reactome: R-HSA-112043), *Ca2+ pathway* (Reactome: R-HSA-4086398), and *Activation of NMDA receptor upon glutamate binding and postsynaptic events* (Reactome: R-HSA-442755).

These processes were supported by oxidative stress-related sources and hubs, including *FDZ4*, *EGFR*, *THRB*, *EP300*, *CAMK2A*, *PRKACG*, *PRKACB*, and *PRKG1*. Literature cites their involvement in both Calcium homeostasis [54–61,92–95], LTP [69,70,80,81,91–96], and ROCC [68–83]. Essential sink nodes involved in both ROCC and cell death through MAPK-JNK signaling include *JUN*, *PPP3CC*, and the *MAPK8* targets *TNF*, *CRK*, *TP53*, and *MAPK11* [70,74–78]. Another remarkable perturbed sink is the *EGFR* [72,73] target phospholipase *PLCB3*, carrying one of the mostly perturbed receptor-target interactions. This is interesting since the well-known involvement of PLCs in the non-canonical WNT/Ca^2+^ signalling pathway [54]. Topologically, the entire FTD-network can be divided into 2 main modules: the non-canonical WNT/Ca^2+^ (Fig 4), and the *PRKACG*-*MAPK8*-*JUN* (Fig 5) sub-networks. Both have a specific role in neuroprotection and neuron survival, respectively, with a critical interface interaction represented by *TCF7L2*-*JUN*-*MAPK8* (Fig 2).

In our model perturbance is a measure of edge perturbation significance deriving from a synthesis of genetic variability (PC1) and phenotype (group) effects, considering the whole network architecture (through SEM). To assess the association between perturbance and FTD-related term enrichment, we used existing annotations to map our network onto FTD-associated KEGG and DO annotations. The KEGG database reports 6 pathways related to FTD (KEGG ID: H00078), including: hsa04010 (*MAPK signaling pathway*), hsa04141 (*Protein processing in the endoplasmic reticulum*), hsa04144 (*Endocytosis*), hsa04310 (*WNT signaling pathway*), hsa04330 (*NOTCH signaling pathway*), and hsa04722 (*Neurotrophin signaling pathway*). As shown in S6 Fig, these pathways encompass the fully-perturbed backbone of our network, suggesting that our method can capture the core FTD-associated variability and extend it through the data-driven concept of edge perturbation. Notably, the FTD-network backbone contains the top-perturbed connections. To have a precise measure of the perturbance content we measured the proportion of perturbed connections in the KEGG FTD-specific sub-network comparing it to the total FTD-network, excluding LVs (i.e. the basal perturbance). The basal perturbance proportion is expected to be already high, since the whole disease-network is associated with FTD: 117/166 (= 70%) significantly perturbed edges. In the KEGG FTD-specific sub-network this percentage is even higher: 40/46 (= 87%). We repeated this measure using DO terms included in the *Nervous System Disease* (DOID:863) and *Disease of Mental Health* (DOID:150) ontology roots, obtaining the DO Nervous System-specific sub-network (S7 Fig). Also in this case, the sub-network perturbance proportion is higher than the basal one: 40/52 (= 77%).

## Discussion

### Biological relevance of the FTD model

In the present study, we investigated whether FTD-associated genetic variability drives the identification of perturbed functional sub-networks, indicating likely impacted biological processes in frontotemporal lobar degeneration. The core structure of the essential FTD-network (Fig 3) highlights the central role of four biological processes in FTD: (i) WNT/Ca^2+^ signaling, (ii) response to oxygen-containing compounds (ROCC), (iii) postsynaptic plasticity and Long Term Potentiation (LTP), and (iv) MAPK-JNK signaling. These functions encompass the entire set of genes and processes defining our theory. As a follow-up to our previous GO-based gene-set analysis [4], several processes appeared replicated, these being neurodevelopment-related processes, Ca^2+^/K^+^ channel regulation and LTP. In addition, in the current study we also found evidence for an implication of oxidative stress and DNA damage, coupled with impairments in the energetic and Ca^2+^/cAMP metabolism, involved in synaptic plasticity and neuroprotection. Particularly, the notion of DNA damage, which is associated with neuronal homeostasis and longevity, appears to gain momentum in the landscape of FTD pathogenesis [81,82,101,102].

Topologically, the FTD-network can be divided into in two main modules: (i) the non-canonical WNT/Ca2+ signaling (ncWNT)-associated module (Fig 4), and (ii) the MAPK/JNK-associated module (Fig 5), including *PRKACG*-*MAPK8*, *AKT3*, and *CREB3L2*, and their sink *JUN*. The interface between the two sub-modules is represented by the *TCF7L2*-*JUN*-*MAPK8* interaction (Figs 2 and 3), depicting an overall disruption of the DAG-IP3/Ca2+/cAMP homeostasis and energetic metabolism, leading to the induction of several oxidative stress-responsive Serine/Threonine (Ser/Thr) kinases and their related targets, including members of the CREB family, protein phosphatases, junction proteins, K^+^ cannels, and members of the FoxO signalling pathway. These critical Ser/Thr kinases include: (a) the Ca^2+^-dependent *CAMK2A*, *PRKCG*, *PRKACG*, *PRKACB*, and *PRKG1*; (b) the MAP-kinases *MAPK8* and *MAPK11*; and (c) the AKT-kinase *AKT3*. Every Ser/Thr kinase has a specific influence over a group of functionally-related nodes, converging towards common target nodes. Specifically, we may define two high-degree sinks (Fig 3): *CTNNB1* and *JUN*. Given these premises, we hypothesized that oxidative stress and DNA damage generated in response to energetic metabolism and Ca^2+^/K^+^ homeostasis impairments, could be among the major potential molecular underpinnings of FTD pathogenesis.

### The non-canonical WNT/Ca^2+^ module

A large portion of our model involves the ncWNT pathway, converging to the sink *CTNNB1*-*CREBBP*, and targeted by a series of perturbed receptors: *ITPR1*-*CAMK2A*, *WNT3*-*FZD4*, *FGF11*-*EGFR*, and *THRB*. In the landscape of WNT signalling, the *WNT3*-*FZD4* interaction might be a novel susceptible functional element in FTD. FZDs are G Protein-Coupled Receptors (GPCRs), involved in downstream DAG-IP3/Ca^2+^/cAMP signalling. WNT/FZD-mediated Phospholipases C (PLCs) activation leads to diacylglycerol (DAG) and inositol triphosphate (IP3) production, stimulating intracellular Ca^2+^ release. Calcium release activates *CAMK2A*, inhibiting the GSK3 kinase and causing nuclear accumulation of beta-catenin (*CTNNB1*) and TCF-mediated gene regulation [54–92]. Our model suggests that the disruption of this mechanism may involve *CAMK2A* directly, as a perturbed (i.e. disease-variant carrying) gene, and through its interaction with *EP300*-*TCF7L2*-*CTNNB1*. Beta-catenin has been implicated in many forms of neurological and cognitive impairments, including the Autism Spectrum Disorder, cell adhesion impairments, dendritic branching, and Long-Term Potentiation (LTP) [103]. Therefore, mutations in the CTNNB1 gene could lead to altered transcriptional activity and impaired synaptic plasticity that may result in brain malformation, intellectual disability, and neuronal loss [104].

*TCF7L2* (a perturbed/seed node) is a beta catenin-interacting transcription factor [63,92] whose involvement in WNT signalling is well documented, although its physiological role in adult brain is still unclear [63]. This supports previous reports about calcium-dependent activation of *CAMK2A* and its implication in neurodevelopment, transcriptional regulation, cell fate determination [54,57,93–96], and neurodegeneration [70]. Furthermore, both *WNT3*-*FZD4* and *FGF11*-*EGFR* interactions reveal a key role of the PLCs *PLCB1*, *PLCB2*, and *PLCB3* within our FTD-network (Fig 4). FTD-associated variants in PLCs may lead to improper intracellular Ca^2+^ levels and thus dysregulation of the Ser/Thr kinases controlling the downstream MAPK-JNK signaling. Previous studies on *PLCB1* showed an association between its depletion and early-onset epileptic encephalopathy [88], suggesting its involvement in learning impairments and neurodegeneration. PLCs activity was recently associated with the activation of the repair factor PARP1. Neuronal DNA is constantly exposed to ROS due to the intense mitochondrial activity in response to the high energy demand required by the Central Nervous System (CNS). This massive exposure to ROS causes the accumulation of single-strand breaks (SSBs) that remain unresolved, despite the presence of repair mechanisms [105]. In particular, aged cerebral neurons showed a group of genes targeted of PARP1-bound SSBs that are implicated in synaptic plasticity and long-term memory.

*CAMK2A* also controls other, peripheral, FTD-relevant targets. In our model, perturbance propagates from *CAMK2A* to calcineurin catalytic subunit *PPP3CC* through calmodulin *CALM2* (Fig 2). Notably, reactive oxygen species (ROS) enhance LTP through calcineurin suppression, inducing cellular and DNA damage, and leading to cell death in neurons [70,80], with constitutively (*as per* genetic association) impaired metabolism. Together with active cell damage, loss of neuroprotection may contribute to neural cell death. *CAMK2A* is connected to three different perturbed routes, involving cAMP and fatty acids metabolism [84–91], and including several mitochondrial proteins. The first route involves the adenylate cyclase *ADCY3*, the phosphodiesterases *PDE1A* and *PDE10A*, and the GMP-deaminase *GMPR* (Fig 2). Several studies highlighted the importance of intracellular levels of cAMP and cGMP for neuroprotection and neuron survival [84–91], and *CAMK2*-dependent *ADCY3* activation has been documented in mouse and rat brain [87]. Specifically, neuron regeneration, survival and synaptic plasticity seem to be enhanced by high intracellular levels of cAMP and cGMP [84–87], and antagonized by phosphodiesterases (PDEs) and myelin-associated glycoproteins (MAGs) [86–88]. In a recent study [88] PDE-dependent cAMP/cGMP control has been documented in Alzheimer’s disease (AD), depression and multiple sclerosis (MS), but not in FTD, to date.

The second *CAMK2*-secific route includes *EP300*, *CPT1B*, and *ACSBG1* (Fig 2). These nodes are involved in fatty acid metabolism and all of them can be found in mitochondria [106–108]. Moreover, *CPT1B* has been recently associated with behavioural disorders characterizing post-traumatic stress both in human and rodent models [107], and *ACSBG1* participate in myelinogenesis [108].

The third *CAMK2*-secific perturbed route is represented by the central network axis *CAMK2A*-*EP300*-*TCF7L2*, showing a bifurcation through *CTNNB1* and *JUN* (Fig 2). We also identified several important junction proteins, such as Ca^2+^, IP3, and cAMP transporters, highlighting the perturbed status of both ncWNT and MAPK signalling pathways. The interaction *PRKCG*-*GJD2*-*TJP1* is one of the perturbation sources acting on *CTNNB1*. *PRKCG* is a Ca^2+^-activated Ser/Thr protein-kinase C (PKC), which mutations are known to be associated with spinocerebellar ataxia, characterized by cognitive impairment, tremor, and sensory loss [109]. Enrichment analysis showed how this PKC is involved in ROCC and LTP. Our model highlights *GJD2* and *TJP1* as possible perturbed *PRKCG* interactors. Former evidences demonstrated a strong association of *GJD2* and *TJP1* with schizophrenia [110].

### Stress-induced Ser/Thr-kinases and neural cell death

The largest sink in our model is *JUN*, and its largest incoming hub is *MAPK8* (aka *JNK1*). The second module of our FTD-network is centred on these two components of the c-Jun N-terminal kinase (JNK) pathway (Fig 5). The two mostly perturbed interactions acting on *JUN* in the entire FTD-network are *PRKG1*-*CREB3L2* (aka *BBF2H7*), and *PRKACG*-*MAPK8*; together with the ncWNT-related route *CAMK2A*-*EP300*-*TCF7L2*.

The cGMP-activated Ser/Thr-kinase *PRKG1* is a key mediator of the nitric oxide (NO)/cGMP signalling pathway, involved in LTP and neuron branching [111]. *CREB3L2*, a *PRKG1* target in the cGMP signalling pathway, is perturbed in our FTD-model. Remarkably, it has been demonstrated that *CREB3L2*, whose expression is induced by endoplasmic reticulum (ER) stress, is involved in preventing the accumulation of unfolded proteins in normal damaged neurons, and protecting neuroblastoma cells from ER-stress induced cell death [112].

On the other hand, *PRKACG* and *MAPK8* are the FTD-connectors with the largest perturbed outgoing connectivity, and sharing one of the mostly perturbed interaction, indicating that they may play a critical role in FTD aetiology. *MAPK8* is a key Ser/Thr kinase capable of phosphorylating *JUN* and prompting stress-induced cell apoptosis by phosphorylating other transcription factors, including *TP53* [74–78], a direct *MAPK8* target. Moreover, our model evidenced a large variety of potentially FTD-associated pro-apoptotic stress-responsive *MAPK8* perturbed interactions and targets. The main target is the disease-variant carrying cytokine *TNF*, sharing with *MAPK8* the second-largest perturbed interaction of the model (after *MAPK8*-*PRKACG*). Other important targets include: the Ser/Thr kinase *MAPK11*, the transcription factor *FOXO1*, the adapter protein *CRK*, the interleukin *IL12B*, the Ca^2+^-dependent phospholipase *PLA2G4B*, and the cytidylyltransferase *PCYT1A*. Conversely, *MAPK8* is influenced by few perturbed interactions, among which the most important is *PRKACG*. The latter is the most degree-central Ser/Thr kinase in the FTD-model after *MAPK8*, and part of the perturbed route *MAPK8-PRKACG-GRIN2B-PRKACB* (Fig 5). *PRKACG* is a protein kinase A (PKA) involved in lipid and glucose metabolism, immune system response, and G1-checkpoint response during cell cycle. It can be dysregulated in response to different kind of stresses, including metabolic stress, DNA damage and cancer [20,78,93,100]. Alongside *PRKACG*, we found other protein kinases, NMDA receptors, and GPCRs that are involved in stress response, (neuronal) apoptosis, and critical neurological functions, such as LTP, learning, and behaviour [69,70,80,81,91–95,99,100]. They include: *PRKACB*, *PRKCG*, *PRKG1*, *AKT3*, *PIK3R1*, *PIK3CA*, *GRIN2B*, together with the EGFR, *CAMK2A*, and *FZD4*. *PRKACG* and *PRKG1* also regulate the activity of the potassium channels *KCNMB4* and *KCNMA1*, respectively, through perturbed interactions. These channels are responsible for membrane excitation and sensitivity to Ca^2+^ levels [113].

### MAPK and NMDA receptor signalling, oxidative stress, and neurodegeneration in FTD

The roots of oxidative damage studies in biology date back to Harman’s free radical theory of aging, in 1954 [68]. The response to oxidative damage involves several different pathways regarding cell fate. We here found that the most ligand-receptors triggering perturbed interactions, control members of the MAPK-JNK signalling pathway. Oxygen radicals, present in ROS, induce phosphorylation of MAPK-related GPCRs or NMDA receptors (NMDARs), activating their coupled PLCs (i.e. *PLCB1*, *PLCB2*, and *PLCB3*), which in turn activate downstream cGMP-dependent kinases, such as *PRKG1*, the Ser/Thr kinases PKA (i.e. *PRKACG*, *PRKACB*), PKC (i.e. *PRKCG*), PKII (i.e. *CAMK2A*), or members of the PI-3K family (i.e. *AKT3* and *PIK3CA*) [57,70,76]. A crucial NMDAR that emerged from our FTD model is *GRIN2B*, part of the NR2 subunit, and acting as glutamate agonist. It is the major excitatory receptor in the mammalian brain. Information exchange through the post-synaptic excitatory CNS neurons occurs mainly through the ionotropic glutamate NMDARs. Synaptic plasticity (i.e. the modulation of the activity of glutamatergic synapses) may persist over long periods of time, leading to the so called LTP. LTP leads to NMDARs activation and influx of Ca^2+^ ions in the post-synaptic membrane. These processes deeply affect key cellular processes for learning and memory [57,70,75].

According to our model, the oxidative stress-responsive Ser/Thr kinase *PRKACG* also influences *MAPK8*. *MAPK8* interacts, in turn, with *MAPK11* (aka p38-beta), a member of p38-MAPK family [75], involved in neuro-inflammatory processes preluding neurodegeneration. This is also supported by the highly perturbation of the osmotic stress-induced cytokine *TNF*, target of *MAPK8* (Fig 5). Furthermore, *MAPK8* may elicit neuronal cell death through activation of the perturbed target *TP53* [73–76]. Another interesting *MAPK8* target is *FOXO1*, involved in oxidative stress response and neuronal cell death [77]. In this respect, our current study supports previous work highlighting a role for oxidative damage and cell degeneration neurological disorders [68–83], including FTD [52,53].

Notably, our findings support neurodegeneration-associated mechanisms, particularly in astrocytes. Astrocytes are glial cells implicated in various neurodegenerative diseases, including Amyotrophic Lateral Sclerosis (ALS), Alzheimer’s Disease (AD), Huntington’s Disease (HD) and Parkinson’s Disease (PD), although not much is known to date in FTD [114]. In general, Astrocyte-mediated onset and progression of these disorders seems to be a consequence of loss of homeostatic functions (e.g., Ca^2+^/cAMP homeostasis, implicated in neuroprotection), and gain of disruptive functions (e.g. abnormal LTP and ROS accumulation) [115,116]. Interestingly, astroglial cells are known to modulate neuronal activity through glutamate release, causing an NMDAR-mediated increase in Ca^2+^ [117,118]. An excess of neuronal metabolic activity, accompanied by an impaired calcium homeostasis, due to astroglial-associated disorders, could make CNS synapses more sensitive to oxidative damage. As reported in the results section, oxygen radicals present in ROS induce phosphorylation of MAPK-associated NMDARs, activating their coupled PLCs, and consequently cGMP-dependent oxygen-responsive kinases (including *PRKG1*, *PRKACG*, *PRKACB*, *PRKCG*, and *CAMK2A*) [57,70,76]. The activity of these oxygen-responsive kinases could be the cause of abnormal LTP, neuroinflammatory response, and neuronal cell death [52,53,68–83].

### Methodological and theoretical aspects

The core of our method consists in reducing the initial network to a minimum set nodes and edges bearing the disease-associated information content of the interactome (KEGG, in this case), filtered to exclude misleading interactions and noise. Although the initial interactome is given, it is then transformed and cross-connected, respectively, by perturbance and covariance-driven LVs inclusion. The last passage enables to also evaluate possible unobserved sources of co-variation. Several disease-relevant aspects apply to our ‘seed-and-expand’ method.

First, our approach assumes that genetic variability influences both single genes and their interaction. We expect that phenotype-associated genetic variants influence proteins’ chemical-physical properties and thus their functions as well as interactions. In this perspective, perturbance is a measure of deviation from the given (i.e. KEGG) interactome. Our supervised-PC1 scores of SNP-to-gene quantification reflects genetic variability at a given locus, therefore the concept of perturbance can be explained in terms of phenotype-associated (i.e. FTD-associated) genetic variation over gene-gene interactions. More in general, the key entity comprised in the definition of perturbance is the information transmitted through such interactions, from the source of perturbation to the final target. If the genotype is altered, also the biochemical properties of the effectors are influenced. Therefore, the genetic alterations that underlie edge perturbations are not limited to deep structural rearrangements, but my also include mild phenotypes that cause small changes in protein functionality and connectivity, resulting in functional impairments [18].

Second, given the scale-free nature of biological regulatory networks [28] one natural class of relevant nodes is represented by hubs, many of which are not perturbed, including *FZD4*, *TJP1*, *EP300*, *JUN*, and *PRKACG* (Figs 2, 3 and 4). This supports the hypothesis according to which hubs are essential for embryonic development and thus are less likely to accumulate disease-related variants that usually turn to be lethal [28,29]. Nevertheless, their functional involvement in the disease phenotype is critical since they send and receive most perturbed connections. However, our method recovered also perturbed hubs, especially those connecting different functional sub-modules, including *TCF7L2* and *MAPK8*. Furthermore, seeds and novel perturbed genes are not necessarily the closest to hubs, but rather those which are mostly influenced by disease-related genetic variation. In our model this is shown through paths maximizing edge perturbation, allowing to avoid the classical and often misleading guilt-by-association rule and favouring disease-relevant (or in general phenotype-relevant) variation. In this scenario, the discovery of novel disease-associated genes is at the same time dependent on gene perturbation, local gene context (i.e. edge perturbation), and the whole disease-network topology, through the overall FTD-model fitting.

The final FTD-network revealed several useful notions: (i) the presence of significantly relevant routes, sources, connectors (including hubs), and targets of perturbance; (ii) the presence of latent “common cause(s)” underling hidden gene-circuits, which connect perturbed routes; (iii) the detection of novel FTD-biomarkers based on functional perturbed connectors. Of note, other than for effect of genetic data, the pipeline is also suited for assessing any kind of quantitative data, including eQTL, expression microarrays, and RNA-seq, in the context of complex disorders.

In conclusion, the current study allowed to expand our previous work [4], revealing the potential functional impact of the genetic variation associated with FTD in the Italian population, using a newly developed network model. Specifically, our study suggests that the genetics underpinning (Italian) FTD is associated with metabolic stress involving possible oxygen compound excess and Calcium/cAMP homeostasis deregulation. This happens through different complex molecular mechanisms, including Adenylate Cyclases, PDEs, stress-induced Ser/Thr-kinases, and WNT/EGFR and NMDA receptors malfunctioning. According to our hypothesis, these impairments lead to neuronal damage, loss of neuroprotection, and eventually to cell death. Moreover, by indicating these complex biological processes we also indicate their key functional players, i.e. those genes that we called novel potential FTD-biomarkers, which appear to be perturbed, and having synergic and small effect size.

## Conclusions

We indicate oxidative stress as a major cause, not only of neurodegeneration in several forms of dementias, but also sporadic FTD. Firstly, we showed as Calcium/cAMP homeostasis and energetic metabolism impairments as the primary causes of loss of neuroprotection and neural cell damage, respectively. Secondly, we described how the altered postsynaptic membrane potentiation due to the activation of stress-induced Serine/Threonine kinases leads to neurodegeneration. By studying the molecular underpinnings of these processes, our study evidences key genes and gene interactions that may account for a significant fraction of unexplained sporadic FTD aetiology.

To the best of our knowledge, this is the first functional network study that highlights potentially impacted biological processes for FTD in the Italian population. In the future, we foresee to apply this method to the entire FTD-GWAS and other population-specific FTD datasets, as well as to the integration of gene expression analysis and genotyping data, to evaluate and refine the impact and translation of disease-associated genetic variation into the functional domain.

## Supporting information

S1 Fig FTD-network. Network obtained by merging the FTD Steiner tree with Structural Equation Model (SEM) covariances, represented as latent variables (L1, L2, …) connecting pairs of bow-free target nodes. The final extracted network is composed by 210 (167 genes + 43 latent variables) nodes and 252 (166 + 2*43) edges. Nodes and edges are labelled according to the conventions followed in Fig 2.
(TIF)

S2 Fig Gene Ontology Biological Process (GO:BP) enrichment dot plot. GO Biological Process enrichment over the 167 FTD-network nodes, displaying the first 40 hits (Bonferroni corrected; p < 1E-9). Every hit is ordered by gene ratio (#enriched_genes/167), and the dot area is proportional to the total number of enriched genes. Coloured dots correspond to the p-value scale, from the lowest (red) to the highest (blue) one.
(TIF)

S3 Fig KEGG enrichment dot-plot. KEGG enrichment analysis over the 167 FTD-module nodes, displaying the first 40 KEGG hits (Bonferroni corrected; p < 1e-5). Every hit is ordered by gene ratio (#enriched_genes/167), and the dot area is proportional to the total number of enriched genes. Coloured dots correspond to the p-value scale, from the lowest (red) to the highest (blue) one.
(TIF)

S4 Fig Reactome enrichment dot-plot. Reactome enrichment analysis over the 167 FTD-module nodes, displaying the first 40 Reactome hits (Bonferroni corrected; p < 0.001). Every hit is ordered by gene ratio (#enriched_genes/167), and the dot area is proportional to the total number of enriched genes. Coloured dots correspond to the p-value scale, from the lowest (red) to the highest (blue) one.
(TIF)

S5 Fig Disease Ontology (DO) enrichment dot-plot. DO enrichment analysis over the 167 FTD-module nodes, displaying the first 23 DO hits (Bonferroni corrected p < 0.002). Every hit is ordered by gene ratio (#enriched_genes/167), and the dot area is proportional to the total number of enriched genes. Coloured dots correspond to the p-value scale, from the lowest (red) to the highest (blue) one.
(TIF)

S6 Fig KEGG FTD-specific sub-network. Sub-network extracted mapping genes annotated with 6 FTD-associated KEGG pathways. It shows a very high density (87%) of perturbed interactions, including the FTD-network backbone. Nodes and edges are labelled according to the conventions followed in Fig 2.
(TIF)

S7 Fig Disease Ontology (DO) nervous system sub-network. Sub-network extracted mapping genes annotated with DO terms descending from *Nervous System Disease* (DOID:863) and *Disease of Mental Health* (DOID:150) roots. It shows a high density (77%) of perturbed interactions, including the FTD-network backbone. Nodes and edges are labelled according to the conventions followed in Fig 2.
(TIF)

S1 Table Maximum Likelihood Estimates (MLE) of the SEM regression parameters. SEM parameter estimation is based on the mean difference between the group variable *C* (0 = control, 1 = case) for each node, adjusted by its parents in the extracted Steiner Tree. Standard errors are calculated by bootstrap (B = 1000 resampling). Significant estimates (p-value < 0.05) are reported in bold.
(DOCX)

S2 Table Selected ancestral bow-free covariances (p < 0.05, values in bold) between pairs of “target” (outgoing degree = 0) nodes of the extracted Steiner tree. Pairs of target genes that do not share a directed path are called ancestral bow-free nodes. When these genes share significant covariances, there may be an unobserved common cause perturbing their interaction. This condition is evaluated using a latent variable (LV) model in which a LV, influenced by the group variable *C* (0 = controls, 1 = cases), is connected to the two bow-free targets. A LV is designed as a significant unknown cause acting on the targets, if the *C*->LV interaction is significant (i.e., p-value(C->LV) < 0.05, in bold), and the LV model has a good fit (i.e., p-value of the Likelihood Ratio Test (LRT) ≥ 0.05, in bold).
(DOCX)
