# Supplementary figures and images for: A novel network analysis approach reveals DNA damage, oxidative stress and calcium/cAMP homeostasis-associated biomarkers in frontotemporal dementia
Source: PLoS One. 2017 Oct 11;12(10):e0185797. doi: 10.1371/journal.pone.0185797 (PMC5636111; doi:10.1371/journal.pone.0185797)

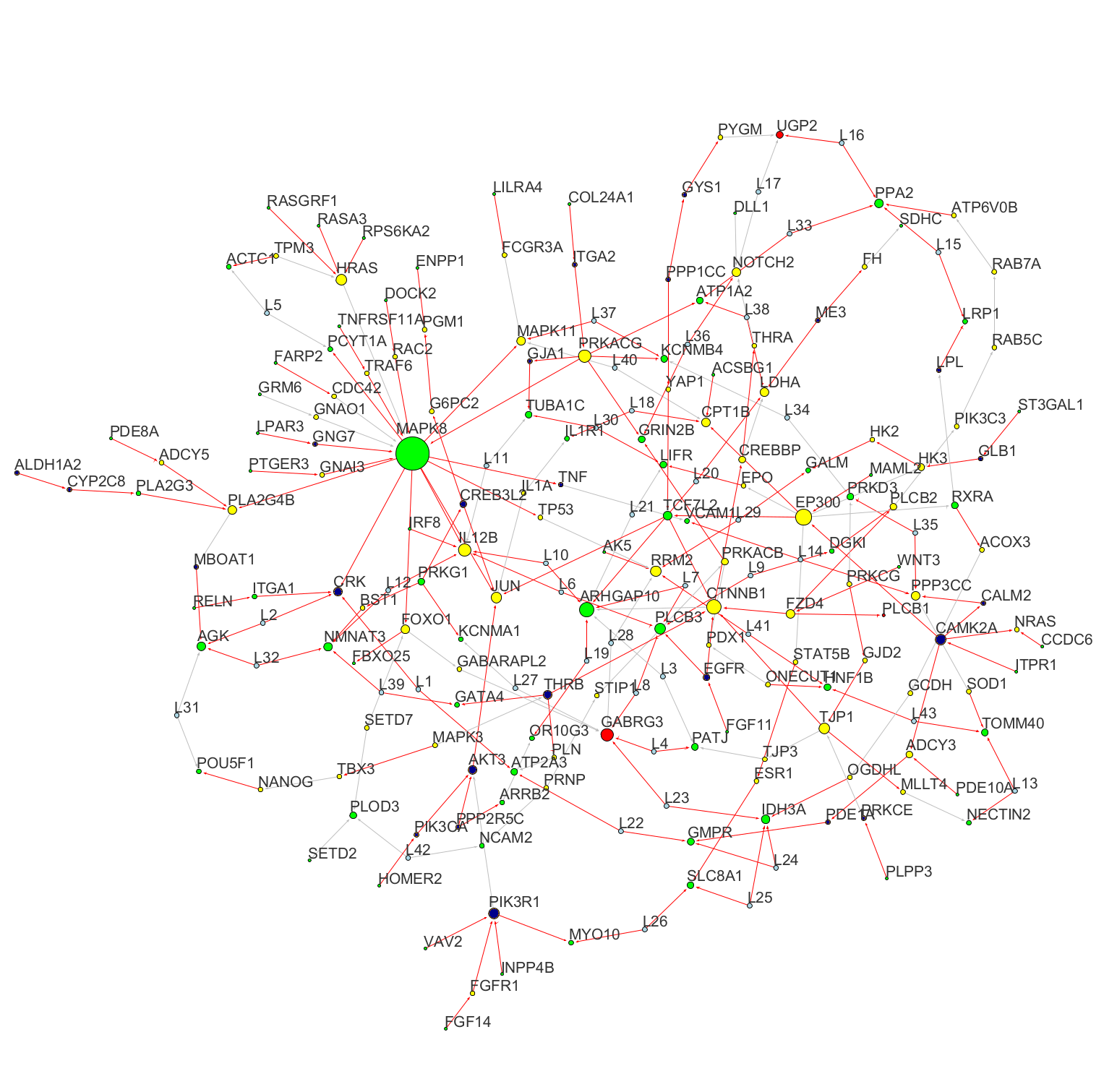

Supplement: S1 Fig — Network obtained by merging the FTD Steiner tree with Structural Equation Model (SEM) covariances, represented as latent variables (L1, L2, …) connecting pairs of bow-free target nodes. The final extracted network is composed by 210 (167 genes + 43 latent variables) nodes and 252 (166 + 2*43) edges. Nodes and edges are labelled according to the conventions followed in Fig 2. (TIF) [file pone.0185797.s001.tif]

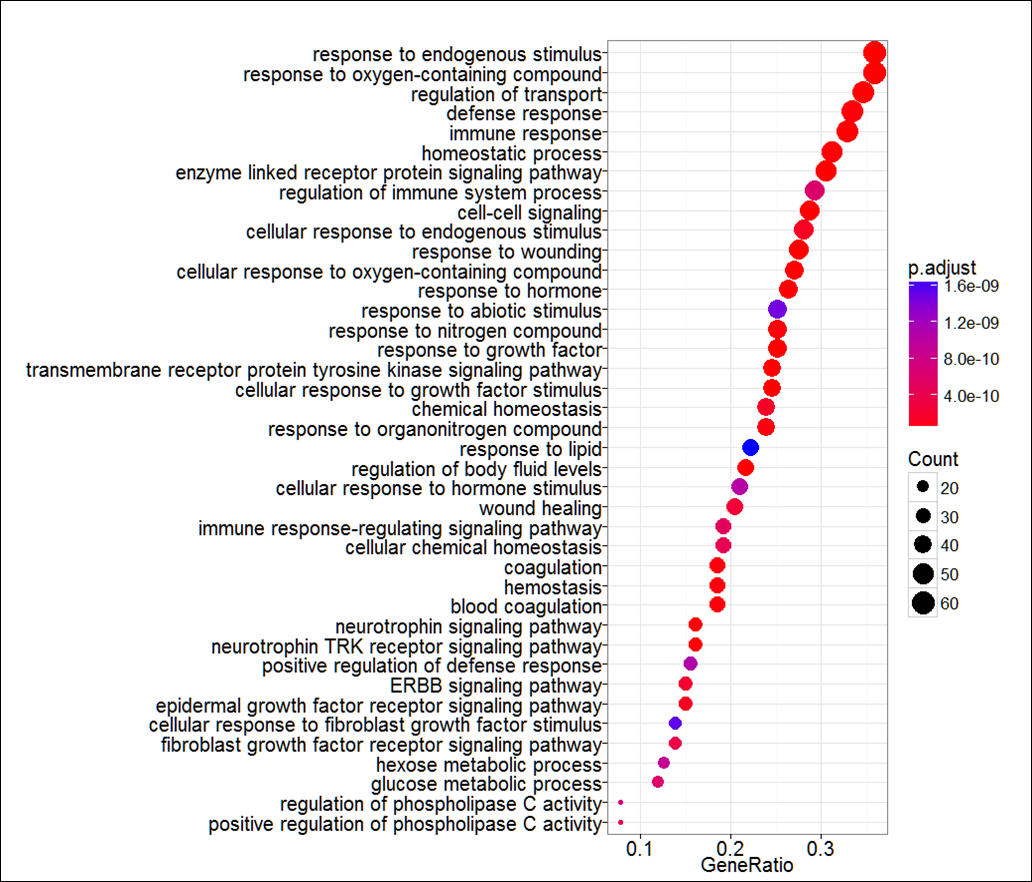

Supplement: S2 Fig — GO Biological Process enrichment over the 167 FTD-network nodes, displaying the first 40 hits (Bonferroni corrected; p < 1E-9). Every hit is ordered by gene ratio (#enriched_genes/167), and the dot area is proportional to the total number of enriched genes. Coloured dots correspond to the p-value scale, from the lowest (red) to the highest (blue) one. (TIF) [file pone.0185797.s002.tif]

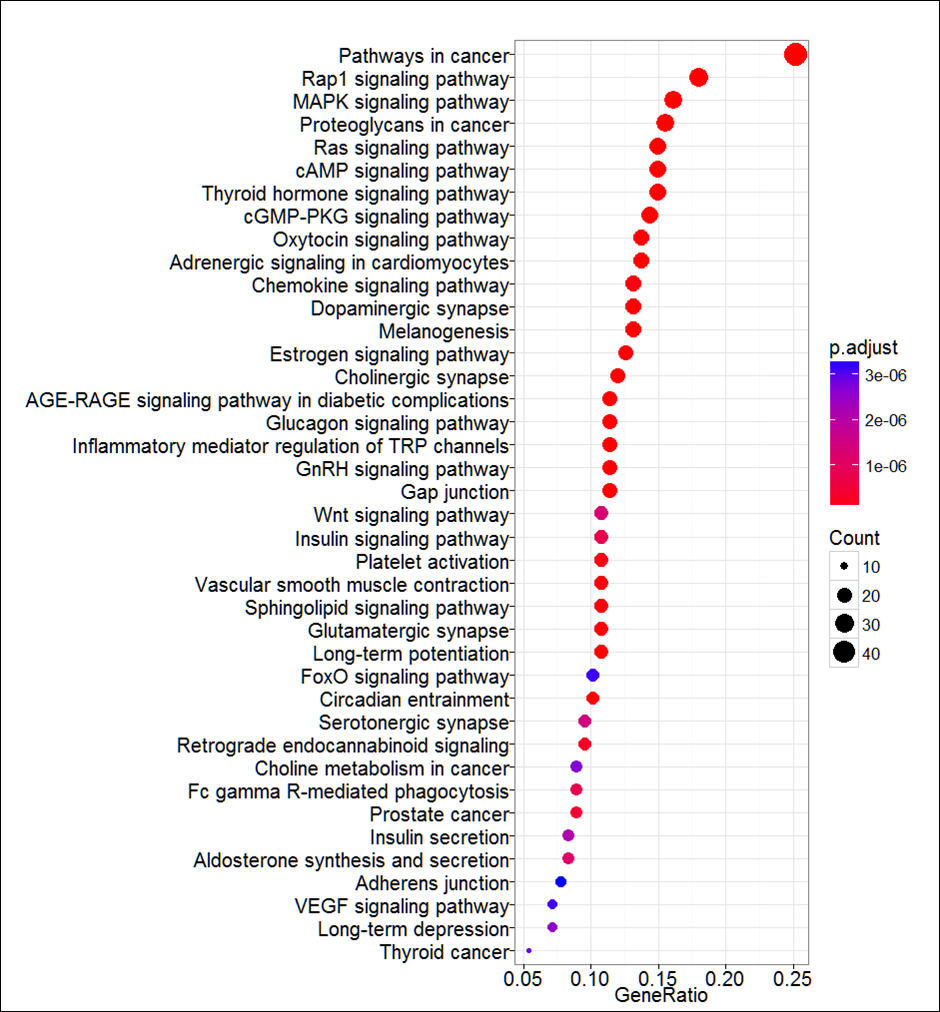

Supplement: S3 Fig — KEGG enrichment analysis over the 167 FTD-module nodes, displaying the first 40 KEGG hits (Bonferroni corrected; p < 1e-5). Every hit is ordered by gene ratio (#enriched_genes/167), and the dot area is proportional to the total number of enriched genes. Coloured dots correspond to the p-value scale, from the lowest (red) to the highest (blue) one. (TIF) [file pone.0185797.s003.tif]

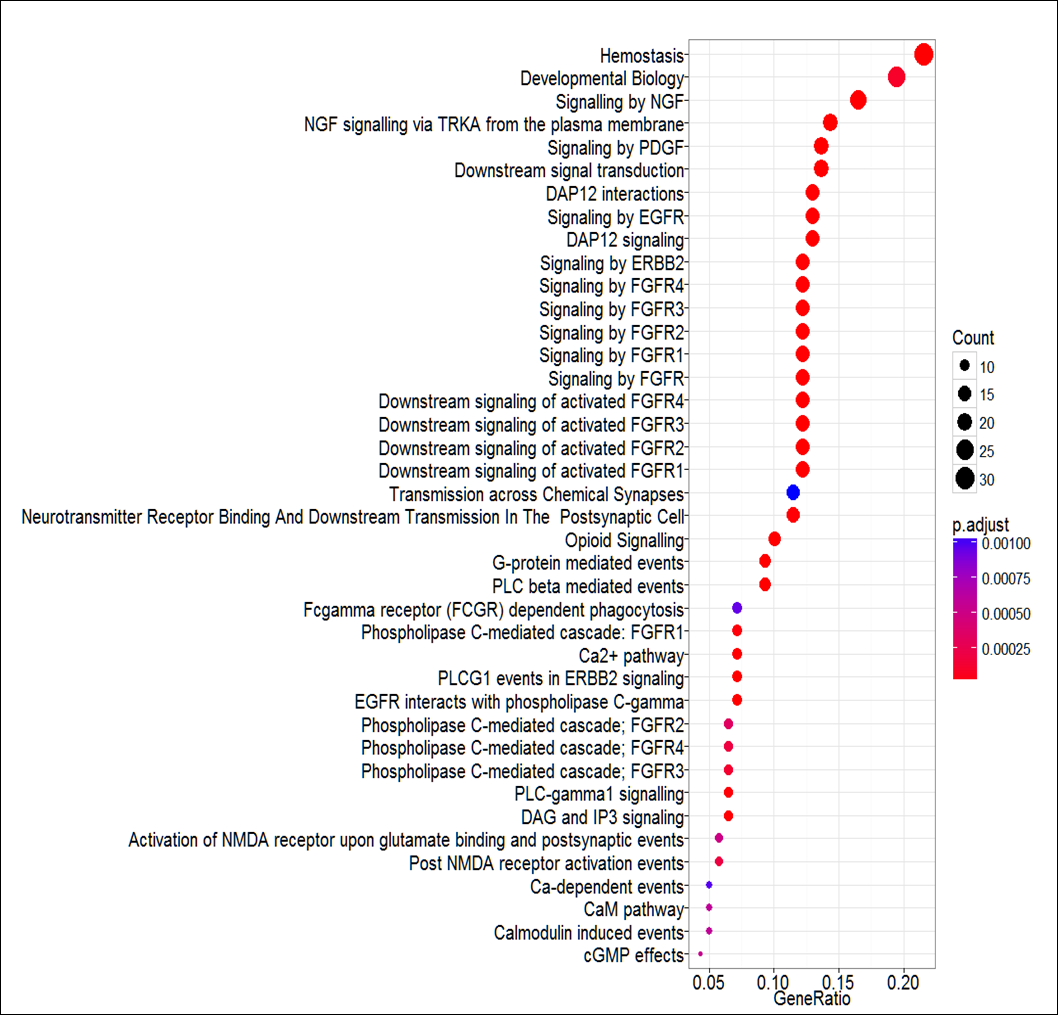

Supplement: S4 Fig — Reactome enrichment analysis over the 167 FTD-module nodes, displaying the first 40 Reactome hits (Bonferroni corrected; p < 0.001). Every hit is ordered by gene ratio (#enriched_genes/167), and the dot area is proportional to the total number of enriched genes. Coloured dots correspond to the p-value scale, from the lowest (red) to the highest (blue) one. (TIF) [file pone.0185797.s004.tif]

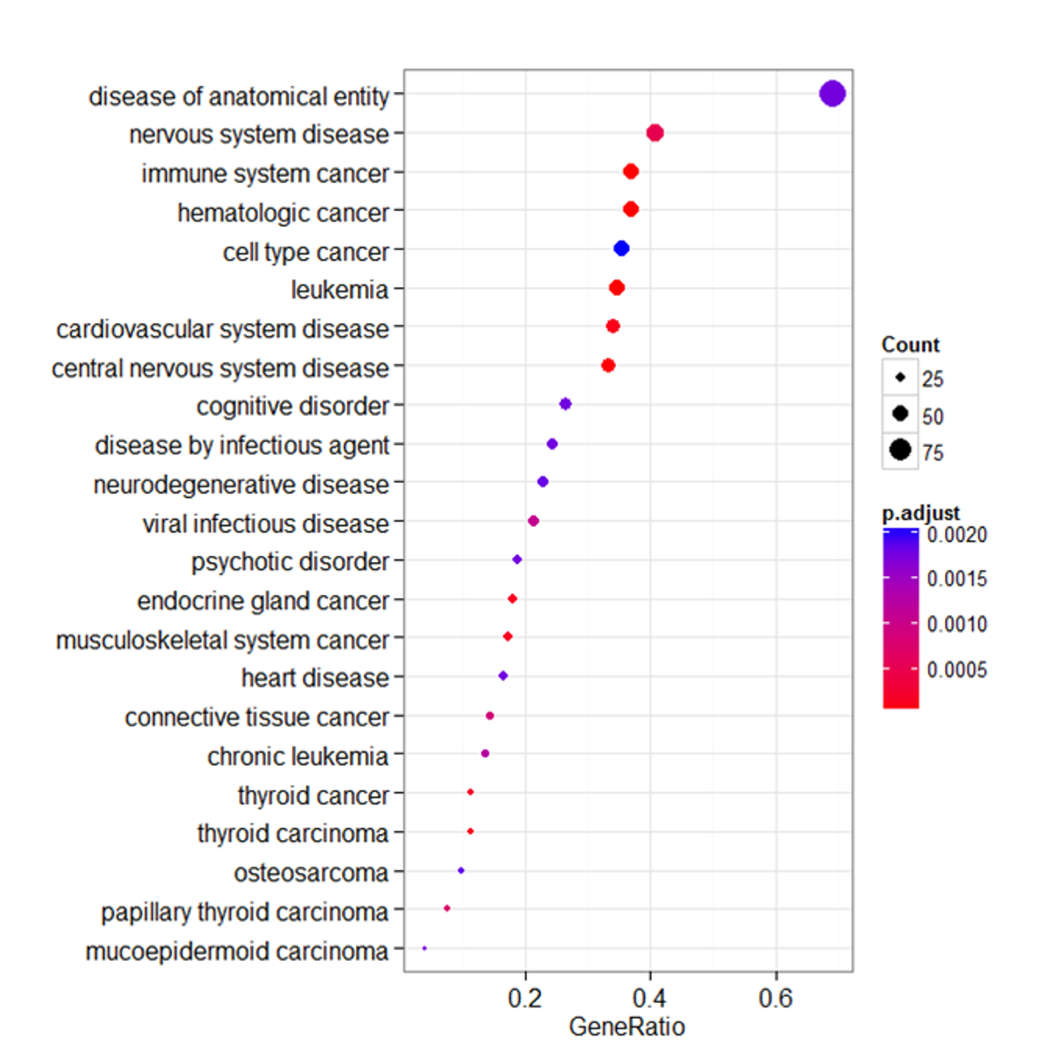

Supplement: S5 Fig — DO enrichment analysis over the 167 FTD-module nodes, displaying the first 23 DO hits (Bonferroni corrected p < 0.002). Every hit is ordered by gene ratio (#enriched_genes/167), and the dot area is proportional to the total number of enriched genes. Coloured dots correspond to the p-value scale, from the lowest (red) to the highest (blue) one. (TIF) [file pone.0185797.s005.tif]

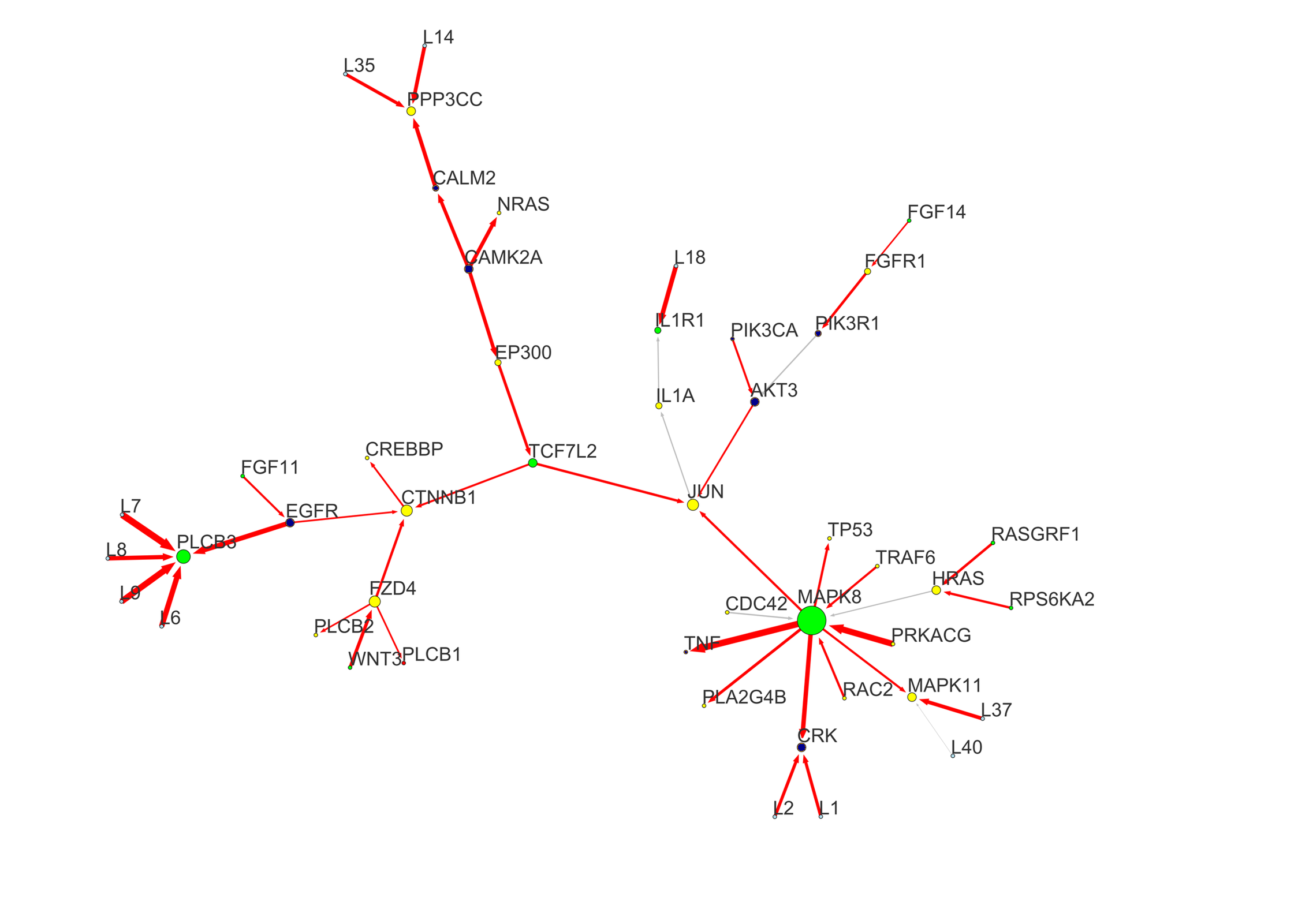

Supplement: S6 Fig — Sub-network extracted mapping genes annotated with 6 FTD-associated KEGG pathways. It shows a very high density (87%) of perturbed interactions, including the FTD-network backbone. Nodes and edges are labelled according to the conventions followed in Fig 2. (TIF) [file pone.0185797.s006.tif]

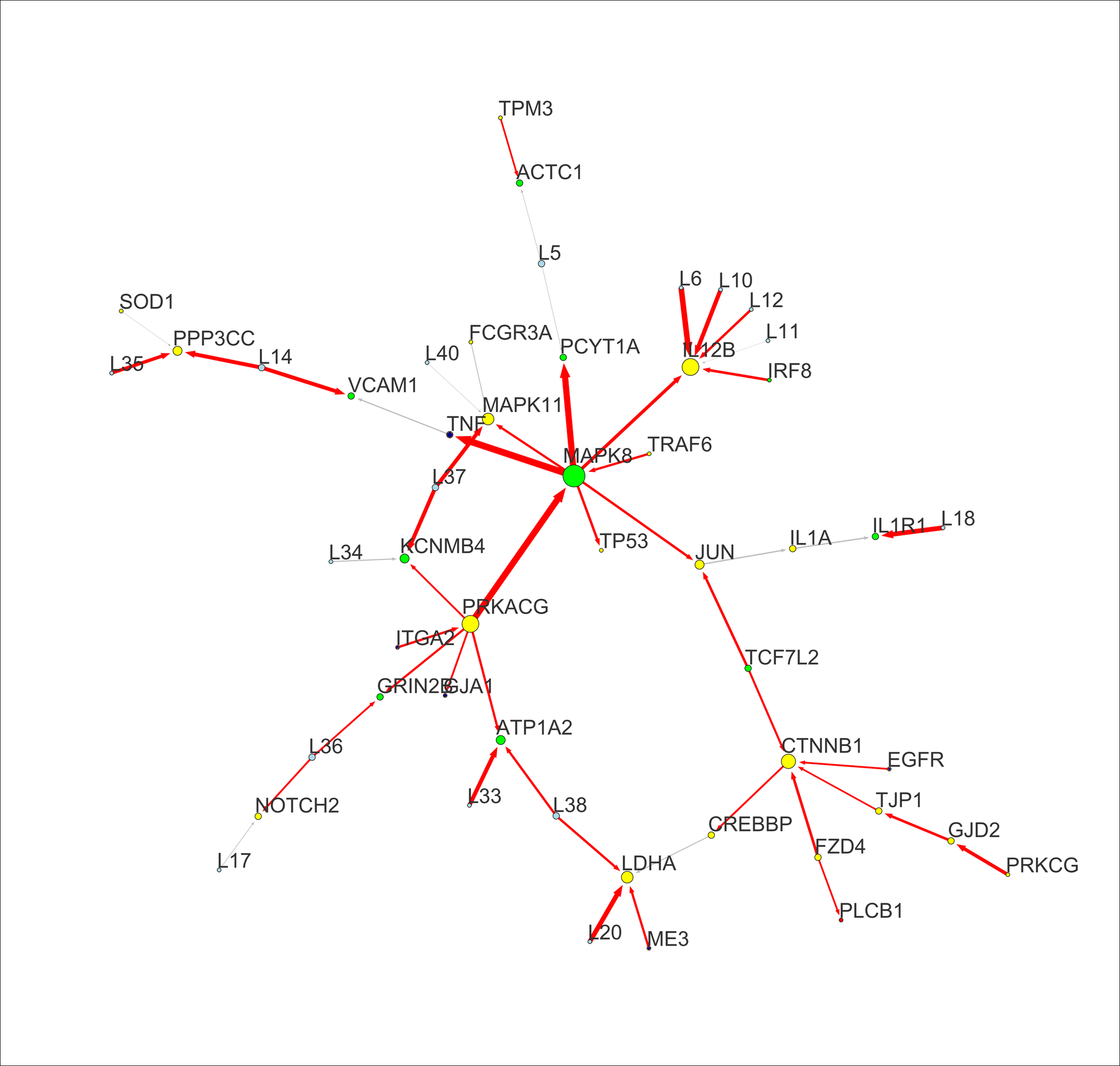

Supplement: S7 Fig — Sub-network extracted mapping genes annotated with DO terms descending from Nervous System Disease (DOID:863) and Disease of Mental Health (DOID:150) roots. It shows a high density (77%) of perturbed interactions, including the FTD-network backbone. Nodes and edges are labelled according to the conventions followed in Fig 2. (TIF) [file pone.0185797.s007.tif]
