# Supplementary material for: A novel network analysis approach reveals DNA damage, oxidative stress and calcium/cAMP homeostasis-associated biomarkers in frontotemporal dementia
Source: PLoS One. 2017 Oct 11;12(10):e0185797. doi: 10.1371/journal.pone.0185797 (PMC5636111; doi:10.1371/journal.pone.0185797)
Supplement: S1 Table — SEM parameter estimation is based on the mean difference between the group variable C (0 = control, 1 = case) for each node, adjusted by its parents in the extracted Steiner Tree. Standard errors are calculated by bootstrap (B = 1000 resampling). Significant estimates (p-value < 0.05) are reported in bold. (DOCX) [file pone.0185797.s008.docx]

**Table S1.** Maximum Likelihood Estimates (MLE) of the SEM regression parameters= mean difference between the group variable C (0=control, 1=case) for each node adjusted by the parents in the extracted Steiner Tree.

| **id** | **genes** | **Estimate** | **p-value** | **p-average** | **seed** |
| --- | --- | --- | --- | --- | --- |
| 38 | PRKG1 | -0.2896347 | **9.95E-08** | **5.47E-05** | YES |
| 9 | PVRL2 | 0.275538796 | **4.01E-07** | 8.94E-02 | YES |
| 165 | HNF1B | 0.251175823 | **3.83E-06** | 3.68E-01 | YES |
| 163 | TOMM40 | -0.24863642 | **4.80E-06** | 3.68E-01 | YES |
| 151 | MYO10 | -0.24697762 | **5.55E-06** | **2.17E-04** | YES |
| 75 | LPAR3 | 0.236821889 | **1.32E-05** | **9.12E-04** | YES |
| 73 | IRF8 | -0.22277993 | **4.17E-05** | **6.80E-03** | YES |
| 89 | ARHGAP10 | -0.22060134 | **4.95E-05** | 1.43E-01 | YES |
| 50 | RASGRF1 | -0.21889975 | **5.66E-05** | **6.07E-03** | YES |
| 36 | PTGER3 | 0.21597954 | **7.10E-05** | **9.67E-03** | YES |
| 6 | PLCB3 | -0.21514035 | **7.58E-05** | **5.60E-05** | YES |
| 43 | LIFR | 0.208412934 | **1.26E-04** | **2.85E-03** | YES |
| 160 | SETD2 | 0.205267036 | **1.60E-04** | 3.68E-01 | YES |
| 48 | RELN | 0.20459013 | **1.68E-04** | **3.61E-06** | YES |
| 29 | IL1R1 | 0.204365663 | **1.71E-04** | 8.92E-02 | YES |
| 44 | TNFRSF11A | 0.20375597 | **1.78E-04** | **4.16E-02** | YES |
| 72 | CCDC6 | -0.20188352 | **2.04E-04** | **1.87E-03** | YES |
| 19 | ITPR1 | 0.200411463 | **2.27E-04** | **1.67E-04** | YES |
| 22 | VCAM1 | -0.20036713 | **2.28E-04** | 1.04E-01 | YES |
| 47 | PDE10A | 0.200110788 | **2.32E-04** | **2.53E-03** | YES |
| 27 | COL24A1 | -0.2001419 | **2.32E-04** | **6.11E-05** | YES |
| 150 | GATA4 | -0.1974709 | **2.81E-04** | **1.44E-04** | YES |
| 24 | PLA2G3 | 0.197358174 | **2.83E-04** | **4.54E-02** | YES |
| 39 | KCNMB4 | 0.196707887 | **2.97E-04** | **3.16E-02** | YES |
| 161 | NCAM2 | 0.192490463 | **3.99E-04** | 3.68E-01 | YES |
| 49 | ITGA1 | 0.192202888 | **4.07E-04** | **9.56E-04** | YES |
| 1 | TCF7L2 | 0.191223269 | **4.36E-04** | **3.13E-02** | YES |
| 91 | ARRB2 | -0.19095103 | **4.44E-04** | 2.47E-01 | YES |
| 69 | MAML2 | 0.190859764 | **4.47E-04** | **1.89E-03** | YES |
| 92 | DOCK2 | -0.1899542 | **4.76E-04** | **9.13E-03** | YES |
| 156 | DLL1 | 0.187900534 | **5.48E-04** | 3.68E-01 | YES |
| 71 | LILRA4 | 0.187558421 | **5.61E-04** | **1.56E-02** | YES |
| 155 | POU5F1 | 0.186665103 | **5.96E-04** | **2.87E-02** | YES |
| 67 | FGF11 | 0.185717946 | **6.35E-04** | **1.63E-02** | YES |
| 158 | PLOD3 | -0.1854397 | **6.47E-04** | 3.68E-01 | YES |
| 93 | PCYT1A | -0.185392 | **6.49E-04** | **7.01E-06** | YES |
| 3 | MAPK8 | -0.18533299 | **6.52E-04** | **2.97E-02** | YES |
| 33 | VAV2 | 0.1848361 | **6.74E-04** | 7.21E-02 | YES |
| 80 | RASA3 | -0.1847337 | **6.79E-04** | **9.37E-03** | YES |
| 59 | ST3GAL1 | 0.183496072 | **7.38E-04** | 1.33E-01 | YES |
| 152 | FBXO25 | -0.18294613 | **7.65E-04** | **2.12E-04** | YES |
| 55 | GRM6 | -0.18224197 | **8.02E-04** | 7.83E-02 | YES |
| 20 | ATP1A2 | -0.1814233 | **8.47E-04** | **1.27E-02** | YES |
| 41 | SDHC | 0.181191827 | **8.60E-04** | 1.08E-01 | YES |
| 57 | AGK | -0.18099797 | **8.71E-04** | **6.45E-03** | YES |
| 15 | ACTC1 | 0.18046665 | **9.02E-04** | 4.17E-01 | YES |
| 34 | RXRA | -0.18033522 | **9.10E-04** | 7.21E-02 | YES |
| 42 | IDH3A | 0.180140299 | **9.21E-04** | **7.69E-03** | YES |
| 70 | OR10G3 | 0.179572204 | **9.56E-04** | 4.94E-01 | YES |
| 79 | PRKD3 | -0.17905926 | **9.89E-04** | 4.09E-01 | YES |
| 11 | FARP2 | 0.17844112 | **1.03E-03** | **5.89E-03** | YES |
| 30 | WNT3 | 0.177551786 | **1.09E-03** | **1.28E-03** | YES |
| 26 | ENPP1 | -0.17711695 | **1.12E-03** | **5.51E-03** | YES |
| 37 | ATP2A3 | 0.177053094 | **1.13E-03** | 4.79E-01 | YES |
| 76 | AK5 | -0.1767397 | **1.15E-03** | 5.52E-02 | YES |
| 148 | INADL | 0.176738332 | **1.15E-03** | 7.19E-01 | YES |
| 64 | RPS6KA2 | -0.17663337 | **1.16E-03** | **9.80E-03** | YES |
| 46 | EGFR | -0.17318269 | **1.44E-03** | **1.68E-02** | NO |
| 12 | ACSBG1 | 0.171810871 | **1.58E-03** | **8.25E-03** | YES |
| 78 | GMPR | -0.17161527 | **1.60E-03** | **5.70E-03** | YES |
| 35 | THRB | -0.17135917 | **1.62E-03** | **4.60E-02** | NO |
| 58 | DGKI | -0.16908299 | **1.87E-03** | **2.15E-03** | YES |
| 51 | HOMER2 | 0.166227933 | **2.23E-03** | **4.38E-02** | YES |
| 126 | PPP2R5C | -0.16591842 | **2.27E-03** | **2.45E-03** | NO |
| 83 | CYP2C8 | 0.165521534 | **2.33E-03** | **3.79E-02** | NO |
| 149 | LRP1 | -0.16244094 | **2.81E-03** | **9.97E-03** | YES |
| 63 | INPP4B | -0.15701851 | **3.87E-03** | **7.46E-03** | YES |
| 88 | CAMK2A | 0.15639545 | **4.02E-03** | **7.42E-03** | NO |
| 5 | PIK3R1 | -0.15513774 | **4.32E-03** | **2.98E-02** | NO |
| 101 | LPL | -0.15340102 | **4.78E-03** | **3.72E-02** | NO |
| 52 | GALM | -0.15101808 | **5.47E-03** | **4.91E-02** | YES |
| 154 | PPA2 | 0.150699297 | **5.57E-03** | **2.04E-02** | YES |
| 77 | PDE8A | -0.15015056 | **5.75E-03** | **4.40E-03** | YES |
| 99 | TNF | -0.14654742 | **7.03E-03** | 5.20E-02 | NO |
| 23 | GRIN2B | 0.142337716 | **8.84E-03** | **2.01E-02** | YES |
| 21 | SLC8A1 | 0.138722303 | **1.07E-02** | **4.62E-02** | YES |
| 94 | PPP1CC | 0.137162388 | **1.16E-02** | **7.76E-03** | NO |
| 60 | GLB1 | 0.136946842 | **1.18E-02** | 7.79E-02 | NO |
| 54 | TUBA1C | -0.13315952 | **1.43E-02** | **9.21E-03** | YES |
| 90 | ITGA2 | -0.13168215 | **1.54E-02** | **6.17E-03** | NO |
| 116 | GJA1 | -0.13111777 | **1.59E-02** | **1.87E-02** | NO |
| 133 | ME3 | 0.128650576 | **1.80E-02** | **2.89E-02** | NO |
| 117 | MBOAT1 | -0.12777152 | **1.88E-02** | **4.29E-02** | NO |
| 127 | AKT3 | -0.12641597 | **2.01E-02** | **2.87E-02** | NO |
| 40 | KCNMA1 | -0.12609205 | **2.04E-02** | **7.66E-08** | YES |
| 81 | DHRS3 | 0.125194982 | **2.13E-02** | **3.05E-03** | YES |
| 10 | PIK3CA | -0.12417587 | **2.24E-02** | **3.01E-02** | NO |
| 134 | PDE1A | -0.1238078 | **2.28E-02** | **1.87E-02** | NO |
| 98 | CREB3L2 | 0.12071971 | **2.64E-02** | **1.36E-02** | NO |
| 68 | NMNAT3 | -0.11857133 | **2.92E-02** | **4.33E-02** | YES |
| 82 | ALDH1A2 | 0.117440334 | **3.08E-02** | **2.34E-03** | NO |
| 66 | FGF14 | 0.117121069 | **3.12E-02** | **4.93E-02** | YES |
| 145 | GYS1 | 0.116881562 | **3.16E-02** | **1.87E-02** | NO |
| 62 | PRKCE | 0.115406554 | **3.38E-02** | **4.48E-02** | NO |
| 112 | CALM2 | 0.115100215 | **3.42E-02** | **1.32E-02** | NO |
| 61 | PPAP2B | -0.11495126 | **3.45E-02** | **2.45E-03** | YES |
| 128 | GNG7 | -0.11344851 | **3.69E-02** | **2.41E-03** | NO |
| 4 | CRK | -0.11242836 | **3.86E-02** | **1.03E-03** | NO |
| 74 | IL12B | -0.10469214 | 5.41E-02 | **4.30E-03** | NO |
| 14 | GJD2 | 0.102364497 | 5.97E-02 | **3.88E-03** | NO |
| 146 | STIP1 | 0.098696432 | 6.95E-02 | 2.49E-01 | NO |
| 85 | CREBBP | -0.0972146 | 7.37E-02 | 7.28E-02 | NO |
| 16 | TPM3 | 0.096387894 | 7.62E-02 | 2.42E-01 | NO |
| 114 | GABARAPL2 | -0.09366034 | 8.49E-02 | 2.93E-01 | NO |
| 7 | TJP1 | -0.09238084 | 8.93E-02 | 5.51E-02 | NO |
| 17 | HRAS | 0.090983196 | 9.42E-02 | **3.82E-02** | NO |
| 97 | ADCY5 | -0.09041468 | 9.63E-02 | **1.08E-02** | NO |
| 108 | ACOX3 | -0.09040508 | 9.63E-02 | **4.85E-02** | NO |
| 86 | PRKACG | -0.08944581 | 9.99E-02 | **1.68E-02** | NO |
| 130 | YAP1 | 0.085493588 | 1.16E-01 | 5.46E-02 | NO |
| 125 | NANOG | 0.079877327 | 1.42E-01 | 7.31E-02 | NO |
| 113 | FOXO1 | -0.07970357 | 1.43E-01 | 1.15E-01 | NO |
| 118 | PGM1 | 0.079342347 | 1.44E-01 | **1.25E-02** | NO |
| 105 | PLN | 0.078446146 | 1.49E-01 | 2.46E-01 | NO |
| 136 | FCGR3A | 0.07817482 | 1.50E-01 | 1.07E-01 | NO |
| 107 | CPT1B | 0.076634725 | 1.59E-01 | **2.13E-02** | NO |
| 31 | FZD4 | 0.07567972 | 1.64E-01 | **2.00E-02** | NO |
| 32 | PLCB2 | 0.074252524 | 1.72E-01 | **3.35E-02** | NO |
| 122 | RAB7A | -0.07383097 | 1.74E-01 | 1.16E-01 | NO |
| 120 | PIK3C3 | -0.07096063 | 1.92E-01 | 2.25E-01 | NO |
| 28 | ADCY3 | 0.070639824 | 1.94E-01 | **1.46E-02** | NO |
| 102 | PYGM | 0.069836615 | 1.99E-01 | **4.70E-02** | NO |
| 162 | PRNP | -0.06972255 | 2.00E-01 | 3.68E-01 | NO |
| 119 | G6PC2 | 0.067242274 | 2.16E-01 | **2.17E-02** | NO |
| 142 | TP53 | 0.06474751 | 2.34E-01 | **3.86E-02** | NO |
| 167 | PDX1 | -0.06192537 | 2.55E-01 | 3.68E-01 | NO |
| 129 | ESR1 | 0.061139892 | 2.61E-01 | **2.36E-02** | NO |
| 95 | EP300 | 0.060310206 | 2.67E-01 | **5.20E-02** | NO |
| 138 | PPP3CC | 0.058559612 | 2.81E-01 | 1.84E-01 | NO |
| 159 | SETD7 | 0.056951167 | 2.95E-01 | 3.68E-01 | NO |
| 103 | IL1A | 0.055179252 | 3.10E-01 | 8.67E-02 | NO |
| 13 | PRKCG | 0.053143131 | 3.28E-01 | 2.05E-01 | NO |
| 53 | HK2 | 0.052667647 | 3.33E-01 | **3.21E-02** | NO |
| 157 | NOTCH2 | 0.04466183 | 4.11E-01 | 3.68E-01 | NO |
| 139 | GNAI3 | -0.04337926 | 4.25E-01 | **6.28E-03** | NO |
| 115 | HK3 | 0.043288478 | 4.26E-01 | **4.87E-02** | NO |
| 18 | PLCB1 | 0.037130577 | 4.95E-01 | **3.48E-02** | YES |
| 100 | MAPK3 | 0.032635523 | 5.48E-01 | 7.17E-02 | NO |
| 104 | THRA | -0.03190196 | 5.57E-01 | 1.95E-01 | NO |
| 25 | UGP2 | 0.031146989 | 5.67E-01 | 5.93E-02 | YES |
| 96 | MLLT4 | 0.031013256 | 5.68E-01 | 5.78E-02 | NO |
| 45 | TRAF6 | -0.03050477 | 5.75E-01 | **2.70E-02** | NO |
| 106 | FH | 0.030447345 | 5.75E-01 | 6.69E-02 | NO |
| 132 | LDHA | 0.02959791 | 5.86E-01 | 1.01E-01 | NO |
| 124 | TBX3 | 0.029444323 | 5.88E-01 | 6.30E-02 | NO |
| 137 | MAPK11 | -0.02892341 | 5.95E-01 | 1.05E-01 | NO |
| 140 | PLA2G4B | -0.02753754 | 6.12E-01 | **2.91E-02** | NO |
| 123 | ATP6V0B | -0.02414505 | 6.57E-01 | **3.73E-02** | NO |
| 153 | GABRG3 | 0.023840031 | 6.61E-01 | 5.06E-01 | YES |
| 84 | CTNNB1 | -0.02183833 | 6.88E-01 | **4.41E-02** | NO |
| 111 | STAT5B | 0.020777336 | 7.02E-01 | **2.68E-02** | NO |
| 8 | TJP3 | 0.020527807 | 7.06E-01 | 4.16E-01 | NO |
| 110 | OGDHL | 0.019978177 | 7.13E-01 | 5.64E-02 | NO |
| 109 | GCDH | -0.01927728 | 7.23E-01 | 1.00E-01 | NO |
| 87 | CDC42 | -0.01908291 | 7.26E-01 | **3.48E-02** | NO |
| 144 | EPO | 0.018667003 | 7.31E-01 | **3.36E-02** | NO |
| 143 | RRM2 | -0.01858068 | 7.33E-01 | 6.16E-02 | NO |
| 121 | RAB5C | -0.0177143 | 7.45E-01 | 2.83E-01 | NO |
| 131 | RAC2 | 0.014967971 | 7.83E-01 | **9.62E-03** | NO |
| 135 | FGFR1 | -0.01402798 | 7.96E-01 | **2.74E-02** | NO |
| 147 | PRKACB | -0.01211706 | 8.24E-01 | 1.74E-01 | NO |
| 141 | BST1 | -0.01081244 | 8.42E-01 | **2.16E-02** | NO |
| 2 | JUN | -0.0100434 | 8.53E-01 | **4.09E-02** | NO |
| 56 | GNAO1 | 0.008870218 | 8.70E-01 | 1.42E-01 | NO |
| 166 | ONECUT1 | 0.007465687 | 8.91E-01 | 3.68E-01 | NO |
| 164 | SOD1 | 0.006381381 | 9.07E-01 | 3.68E-01 | NO |
| 65 | NRAS | -0.00552796 | 9.19E-01 | **1.22E-03** | NO |
|  |  |  |  |  |  |

*Legend:* Estimate= MLE of the parameter, p-values= p-values of the t-test=estimate/bootstrap standard error with B=1000 bootstrap resamplings, p-average= average weight (i.e. p-value) of the neighbourhood for each node, seed= if node is a seed gene
