# Supplementary material for: A novel network analysis approach reveals DNA damage, oxidative stress and calcium/cAMP homeostasis-associated biomarkers in frontotemporal dementia
Source: PLoS One. 2017 Oct 11;12(10):e0185797. doi: 10.1371/journal.pone.0185797 (PMC5636111; doi:10.1371/journal.pone.0185797)
Supplement: S2 Table — Pairs of target genes that do not share a directed path are called ancestral bow-free nodes. When these genes share significant covariances, there may be an unobserved common cause perturbing their interaction. This condition is evaluated using a latent variable (LV) model in which a LV, influenced by the group variable C (0 = controls, 1 = cases), is connected to the two bow-free targets. A LV is designed as a significant unknown cause acting on the targets, if the C->LV interaction is significant (i.e., p-value(C->LV) < 0.05, in bold), and the LV model has a good fit (i.e., p-value of the Likelihood Ratio Test (LRT) ≥ 0.05, in bold). (DOCX) [file pone.0185797.s009.docx]

Table S2. Selected ancestral bow-free covariances (p<0.05) between pairs of “target” (outgoing degree=0) nodes of the extracted Steiner Tree.

| **id** | **gene (j)** | **gene (k)** | **covariance** | **p-value (LC)** | **p-value (LRT)** |
| --- | --- | --- | --- | --- | --- |
| 15 | LRP1 | PPA2 | -0.06380048 | **0.000592866** | **0.99858622** |
| 24 | IDH3A | GMPR | -0.07225923 | **0.000113884** | **0.88093182** |
| 12 | IL12B | NMNAT3 | 0.06412931 | **0.009040237** | **0.87756553** |
| 22 | ATP2A3 | GMPR | -0.06560328 | **0.000160631** | **0.84972458** |
| 17 | UGP2 | NOTCH2 | 0.05369545 | 0.314815908 | **0.80772562** |
| 33 | PPA2 | ATP1A2 | -0.06954391 | **0.000371332** | **0.63166788** |
| 28 | GABRG3 | RRM2 | 0.06215951 | 0.942256483 | **0.57131294** |
| 39 | GATA4 | NMNAT3 | 0.08189078 | **0.000179489** | **0.47060246** |
| 26 | MYO10 | SLC8A1 | -0.08062742 | **5.88E-06** | **0.428216** |
| 13 | NECTIN2 | TOMM40 | -0.91151426 | **4.96E-07** | **0.41213359** |
| 1 | CRK | ATP2A3 | -0.06387032 | **0.002074717** | **0.38852055** |
| 30 | TUBA1C | LIFR | -0.06720461 | **0.000278691** | **0.36109928** |
| 2 | CRK | AGK | 0.06066072 | **0.001896435** | **0.36053018** |
| 35 | PRKD3 | PPP3CC | -0.05497548 | **0.00156186** | **0.31263659** |
| 14 | VCAM1 | PPP3CC | -0.06330877 | **0.000578183** | **0.22544657** |
| 27 | GABRG3 | KCNMA1 | -0.05420102 | 0.066655154 | **0.20984706** |
| 10 | IL12B | ARHGAP10 | 0.07303581 | **0.000307228** | **0.18670105** |
| 40 | CPT1B | MAPK11 | 0.06857948 | 0.511612773 | **0.15918687** |
| 16 | UGP2 | PPA2 | 0.05598574 | **0.028849926** | **0.15122641** |
| 29 | GALM | RRM2 | 0.0615048 | **0.017142169** | **0.1421398** |
| 23 | IDH3A | GABRG3 | 0.0572429 | **0.002346414** | **0.12994212** |
| 20 | ARHGAP10 | LDHA | -0.06028625 | **0.000123758** | **0.08838541** |
| 38 | LDHA | ATP1A2 | -0.06018741 | **0.011822519** | **0.06213609** |
| 8 | PLCB3 | GABRG3 | -0.06995237 | **0.000205395** | **0.05646796** |
| 37 | KCNMB4 | MAPK11 | 0.05187299 | **0.000587149** | 0.04227176 |
| 4 | PATJ | GABRG3 | -0.05698216 | **0.002887498** | 0.0387468 |
| 36 | GRIN2B | NOTCH2 | -0.05431595 | **0.029821392** | 0.02694754 |
| 18 | IL1R1 | CPT1B | -0.04952199 | **0.000116319** | 0.0145007 |
| 41 | RRM2 | HNF1B | -0.05449643 | **0.001485604** | 0.00972141 |
| 25 | IDH3A | SLC8A1 | -0.04800161 | **0.000164586** | 0.00261847 |
| 32 | AGK | NMNAT3 | -0.05705745 | **0.000312227** | 0.00146762 |
| 6 | PLCB3 | IL12B | -0.06009428 | **2.36E-05** | 0.00138873 |
| 11 | IL12B | TUBA1C | -0.06885976 | 0.624924444 | 0.00088872 |
| 7 | PLCB3 | ARHGAP10 | -0.04347468 | **1.63E-06** | 0.00068928 |
| 9 | PLCB3 | DGKI | -0.052803 | **4.08E-06** | 0.00049606 |
| 19 | ARHGAP10 | OR10G3 | 0.05348135 | **3.13E-06** | 0.00036776 |
| 5 | ACTC1 | PCYT1A | 0.05209135 | 0.411584217 | 1.59E-06 |
| 34 | PRKD3 | KCNMB4 | 0.04461387 | 0.136024856 | 1.03E-06 |
| 42 | PLOD3 | NCAM2 | 0.05480919 | 0.241200468 | 8.16E-07 |
| 31 | AGK | POU5F1 | 0.06504805 | 0.517237126 | 6.21E-07 |
| 3 | PATJ | ARHGAP10 | 0.05837258 | 0.069915032 | 2.08E-07 |
| 21 | ARHGAP10 | LIFR | 0.05755647 | 0.337647018 | 8.55E-09 |
| 43 | TOMM40 | HNF1B | 0.04489405 | 0.021170893 | 1.29E-10 |
|  |  |  |  |  |  |

*Legend:* p-value(LC)= bootstrapped p-value (B=1000 bootstrap resamplings) of the edge C->L fitting a LV model in which two target genes are connected through a LV of the underlying common unknown cause(s) acting on them, and the LV is related to group *C* (1=case; 0=control); p-value (LRT) = p-value of the goodness of fit Likelihood Ratio Test (LRT) for the LV model (good fit if p > 0.05).
